# Alterations in Leg Muscle Glucose Uptake and Inter-Limb Asymmetry after a Single Session of tDCS in Four People with Multiple Sclerosis

**DOI:** 10.3390/brainsci11101363

**Published:** 2021-10-16

**Authors:** Alexandra C. Fietsam, Justin R. Deters, Craig D. Workman, Laura L. Boles Ponto, Thorsten Rudroff

**Affiliations:** 1 Department of Health and Human Physiology, University of Iowa, Iowa City, IA 52242, USA; alexandra-fietsam@uiowa.edu (A.C.F.); justin-deters@uiowa.edu (J.R.D.); craig-workman@uiowa.edu (C.D.W.); 2 Department of Radiology, University of Iowa Hospitals and Clinics, Iowa City, IA 52242, USA; laura-ponto@uiowa.edu; 3 Department of Neurology, University of Iowa Hospitals and Clinics, Iowa City, IA 52242, USA

**Keywords:** tDCS, neuroimaging, positron emission tomography, walking, asymmetries, multiple sclerosis

## Abstract

Asymmetrical lower limb weakness is an early symptom and significant contributor to the progressive worsening of walking ability in people with multiple sclerosis (PwMS). Transcranial direct current stimulation (tDCS) may effectively increase neural drive to the more-affected lower limb and, therefore, increase symmetrical activation. Four PwMS (1 female, age range: 27–57) underwent one session each of 3 mA or SHAM tDCS over the motor cortex corresponding to their more-affected limb followed by 20 min of treadmill walking at a self-selected speed. Two min into the treadmill task, the subjects were injected with the glucose analog [^18^F]fluorodeoxyglucose (FDG). Immediately after treadmill walking, the subjects underwent whole-body positron emission tomography (PET) imaging. Glucose uptake (GU) values were compared between the legs, the spatial distribution of FDG was assessed to estimate glucose uptake heterogeneity (GUh), and GU asymmetry indices (AIs) were calculated. After tDCS, GU was altered, and GUh was decreased in various muscle groups in each subject. Additionally, AIs went from asymmetric to symmetric after tDCS in the subjects that demonstrated asymmetrical glucose uptake during SHAM. These results indicate that tDCS improved GU asymmetries, potentially from an increased neural drive and a more efficient muscle activation strategy of the lower limb in PwMS.

## 1. Introduction

One of the first signs of multiple sclerosis (MS), an inflammatory autoimmune disease of the central nervous system (CNS) [1], is weakness in one limb, particularly in the lower limbs. The resulting lower limb strength asymmetry is a significant contributor to the progressive worsening of walking abilities, which is considered to be one of the most important factors leading to decreased quality of life in people with multiple sclerosis (PwMS) [2,3]. However, treatments to correct strength asymmetries and improve walking performance in PwMS are currently ineffective and improved treatments are highly warranted.

Transcranial direct current stimulation (tDCS) uses weak currents applied to the scalp to non-invasively alter cortical excitability [4] and is considered safe with only mild transient adverse side effects (e.g., temporary tingling sensations) [5,6]. It is generally accepted that anodal tDCS enhances cortical excitability [7], and cathodal tDCS decreases cortical excitability (inhibition) [8]. Therefore, tDCS may effectively increase cortical excitability and enhance neural drive from the cortex to the spinal motor neuron pool, particularly in patients with disease characterized by hypo-excitability of motor areas, such as PwMS. Previous studies have demonstrated improved motor performance after tDCS in healthy subjects [9], older adults [10], and various neurological populations [11,12], including PwMS [13,14,15,16]. However, variability in the severity and symptomology of MS might lead to inconsistencies in tDCS study results. Indeed, MS symptoms are unpredictable and vary in type and severity between patients and within the same patient over time [17]. Additionally, symptoms may disappear or remit completely in some PwMS, while they may persist or even worsen over time for others [17].

It is well accepted that glucose is the main energy source for exercising skeletal muscles [18]. Thus, positron emission tomography (PET) imaging using [^18^F]fluorodeoxyglucose (FDG), a glucose analogue, is a feasible approach to objectively evaluate the activity of multiple lower limb muscles during physical activity and circumvent the disadvantages inherent to surface EMG (e.g., inability to evaluate deep-lying muscles [18,19]). Increased glucose uptake (GU) and GU asymmetry between the same muscles of different legs have been associated with a greater metabolic cost of walking, increased energy demands [20,21,22], and early fatigability [2,23] in PwMS. This is because a disproportionate loss of lower threshold (less-fatigable) motor units is commonly seen in PwMS and can lead to a compensatory increase in the activity of higher threshold (fast-fatigable) motor units that consume more glucose and fatigue more rapidly [24,25]. Therefore, FDG-PET might identify which skeletal muscles are compromised and might be contributing to the impaired walking performance and greater fatigability of PwMS. Importantly, FDG-PET might also provide information about muscle fiber (i.e., motor unit) activity by investigating the spatial distribution of FDG, using the coefficient of variation, within individual muscles to estimate glucose uptake heterogeneity (GUh) [26,27,28]. Specifically, as the number of recruited MUs increases, the proportion of activated muscle fibers also increases and GUh declines [26].

Importantly, tDCS group outcomes may not reflect individual patient responses, which might have a negative impact on our understanding of the pathophysiology, treatment, and management of diseases such as MS. Therefore, and given the variabilities inherent to both MS and tDCS, studies reporting the individual responses of a small number of patients could be useful for determining which stimulation parameters (e.g., brain target, intensity, timing) may be effective for people specific symptomology. Additionally, as suggested by Abu-Zidan et al. [29], four patients or less should be reported individually as case reports. Therefore, the purposes of this study were (1) to examine the effects of a single session of 3 mA tDCS applied over the motor cortex representation of the more-affected leg on GU and GU asymmetry during treadmill walking in PwMS and (2) to investigate if changes in GUh might explain the improved muscle activation asymmetries. Our primary hypothesis is that tDCS would decrease asymmetry between the leg muscles by inducing greater muscle activation in the more-affected leg, as indicated by altered glucose uptake. Additionally, considering changes in skeletal muscle properties and central activation in PwMS [25,30,31], we theorized that PwMS do not efficiently recruit motor units during walking and instead rely on already recruited muscle fibers (motor units) for movement, which results in greater GUh. Therefore, our secondary hypothesis is that PwMS would have greater GUh in SHAM, especially in the more-affected leg, and that tDCS would decrease GUh within individual muscles. Here, we present the effects of tDCS and SHAM stimulation administered before treadmill walking on leg muscle glucose uptake in four PwMS. 

## 2. Materials and Methods

### 2.1. Participants

The inclusion and exclusion criteria utilized for this protocol are identical to a previous case report from this lab [32]; a brief description is provided here. The subjects were included if they were between 18–70 years of age, had a positive relapsing-remitting MS diagnosis from a neurologist in accordance with the 2017 revised McDonald’s criteria [33], self-reported leg-strength asymmetry, and were able to walk for 20 continuous minutes. Exclusion criteria were an MS relapse in the previous 60 days, unable to fast for 6 h, high risk for cardiovascular disease per the American College of Sports Medicine risk classification system [34], modifications to disease-altering medications within the last 45 days, any concurrent neurological disorder or disease, hospitalization within the previous 90 days, pregnancy, seizure history, or on medications known to lower the seizure threshold, and inability to sign or understand the informed consent form. The study was conducted in accordance with the Declaration of Helsinki and was approved by the Institutional Review Board at the University of Iowa. Each participant signed informed consent before participating.

### 2.2. Experimental Protocol

**Figure 1:**
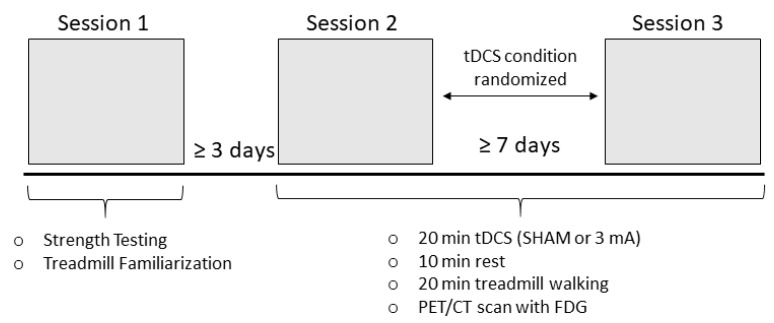
Experimental protocol. Subjects attended three total sessions. During Session 1, subjects completed isokinetic strength testing to determine their more-affected leg and self-selected their treadmill walking speed for subsequent sessions. During Session 2 and 3, subjects underwent 20 min of either SHAM or 3 mA transcranial direct current stimulation (tDCS (determined through randomization) over the motor cortex area representing their more-affected leg, a 10-min rest, then 20 min of treadmill walking. After the walking task, the subjects underwent a positron emission tomography/computed tomography (PET/CT scan with fluorodeoxyglucose (FDG)).

This study utilized a single-blind, randomized, SHAM-controlled, cross-over design. Each participant attended three experimental sessions, with sessions 1 (strength testing and treadmill familiarization) and 2 (first tDCS/PET Scan session) separated by at least 3 days to allow for ample recovery after strength testing [35,36] and sessions 2 and 3 (first and second tDCS/PET sessions) separated by at least 7 days to allow the tDCS effects to subside [7,37]. The experimental protocol is shown in Figure 1. During Session 1, subjects were consented and then filled out the Patient Determined Disease Scale (PDDS), which is strongly correlated with and is considered an alternate assessment to the Expanded Disability Disease Status Scale (EDSS) [38] and the fatigue severity scale (FSS) questionnaires. The subjects then completed isokinetic strength testing to objectively determine their more-affected (weaker) leg. Subsequently, the subjects walked on a treadmill to self-select a comfortable walking pace, which was utilized in experimental sessions 2 and 3. At the beginning of Session 2 and 3, blood glucose, height, and weight were measured, and an IV catheter was inserted to facilitate FDG administration. Prior to Sessions 2 and 3, the subjects fasted for a minimum of 6 h and blood glucose was required to be ≤ 200 mg/dL to proceed with FDG administration and the PET scanning [39,40]. The subjects then sat comfortably in a chair and received 20 min of SHAM or tDCS (3 mA; stimulation condition was randomized) targeting the motor cortex corresponding to their more-affected leg, as determined in the strength testing (see below). The subjects then rested for 10 min to allow for optimal stimulation effects [41,42]. After this rest period, the subjects walked on a treadmill for 20 min at the speed determined in Session 1. Two minutes into the walking, ~10 ± 10% mCi of FDG was injected via IV injection. Immediately after the 20-min walking task was completed, the subjects underwent positron emission tomography/computed tomography (PET/CT) imaging. 

### 2.3. Strength Testing

The strength testing was performed on an isokinetic dynamometer (HUMAC NORM, CSMi, Stoughton, MA, USA). The subjects began the testing with a warm-up of the knee extensors and flexors consisting of 15 submaximal repetitions of concentric/concentric contractions at 60°/s. Once completed, the subjects rested for ≥ 30 s before the maximal strength testing protocol began. The strength assessment consisted of five repetitions of maximal effort knee extension and flexion (concentric/concentric) at 60°/s. The testing was performed on the right leg first, followed by the left leg after a 2-min rest. Vigorous verbal encouragement was provided to encourage a maximal effort during each repetition. The maximum torque produced by each leg was utilized to objectively determine the more-affected leg [5,13]. 

### 2.4. Transcranial Direct Current Stimulation

Stimulation was delivered via two carbon electrodes (Soterix Medical Inc., New York, NY, USA) inserted into 5 cm × 7 cm saline-soaked sponges. The anode was placed over the motor cortex (C3 or C4, using the 10–20 electroencephalography (EEG) placement convention [43]) representing the more-affected leg, with electrode oriented at a 45° to the coronal plane to ensure alignment with the motor cortex and the medial edge covering the center of the skull (Cz) [44]. The cathode was placed on the contralateral supraorbital area. This montage has been commonly used to maximize motor performance and was chosen to target the more-affected M1 [45,46,47]. During the active condition, the device ramped up over 30 s to the target intensity of 3 mA, where it remained for 20 min. At the end of this 20 min period, the current ramped down to 0 mA over 30 s. This protocol has been proven effective at increasing cortical excitability for > 30 min [48]. During SHAM, the current ramped up to 3 mA over 30 s then immediately ramped down to 0 mA over a 30 s period at the beginning and end of the 20-min stimulation period, but otherwise remained at 0 mA. Although most studies use stimulation intensities ≤ 2 mA, higher intensities may be required to significantly alter cortical excitability [49], particularly in people with diseases characterized by decreased cortical excitability, such as PwMS. Moreover, several studies have demonstrated that intensities up to 4 mA are safe, tolerable, and do not elicit any serious adverse effects [5,50,51,52]. 

To evaluate the tolerability and blinding, each subject was asked to report all sensations felt during the stimulation period [6]. Specifically, the subjects were asked to rate the severity of sensations felt on a 10-point Likert scale (1 = “barely perceptible”; 10 = “most I could possibly stand”). For blinding, the subjects were asked whether they thought they received SHAM or active stimulation and to rate their confidence in their guess on a similar 10-point Likert scale (1 = not confident at all; 10 = completely confident). Their responses were recorded, but blinding integrity was maintained until after their final session was completed. 

### 2.5. PET/CT Imaging

Two minutes into the 20-min walking task, ~10 mCi of FDG was injected via IV. This timing allowed for subjects to reach a steady-state before injection and sufficient time for glucose uptake into the muscles before imaging [53]. After the 20-min walking task was completed, the subjects were immediately led to the PET/CT scanner for image acquisition. CT scans were acquired prior to PET acquisition to provide anatomical and attenuation correction information. The subjects were secured in place on the same scanning table for both scans to allow for co-registration of the PET and CT scans. The scans were collected with a GE Discovery MI Time of Flight PET/CT scanner with SiPM array detector technology (GE Healthcare, Waukesha, Wisconsin). The whole body was scanned with a scan duration between 2 and 5 min in length per bed position, depending on the BMI of the subject, and was followed by 5 min per bed position for the lower limbs. All data sets were reconstructed via OSEM 3D reconstruction with 16 subsets and 3 iterations along with a Gaussian smoothing of 5 mm. Additionally, all data sets were corrected for scatter, random coincidence, and dead-time.

### 2.6. Data Analysis

**Figure 2:**
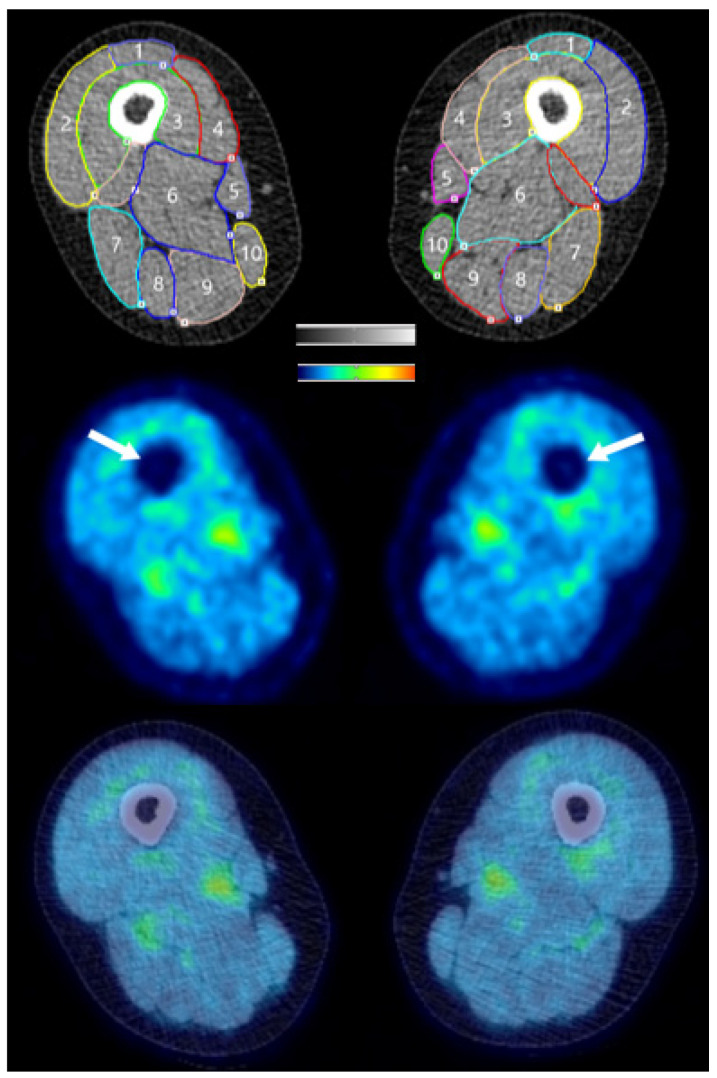
Cross-sectional slice of a CT scan taken at the mid-thigh level in Subject 4. Regions of interest (ROIs) were drawn on the computer tomography (CT) scan (top image) to indicate the different skeletal muscles of the lower limb. 1 = Rectus Femoris, 2 = Vastus Lateralis, 3 = Vastus Intermedius, 4 = Vastus Medialis, 5 = Sartorius, 6 = Adductor Magnus (not included in the analysis), 7 = Biceps Femoris, 8 = Semitendinosus, 9 = Semimembranosus, 10 = Gracilis. The ROIs were copied to the corresponding positron emission tomography (PET) image (middle image). The white arrows indicate the femur in the middle image. The bottom image is an example of a PET scan co-registered to a CT scan. Red denotes the greatest signal intensity, followed by yellow, green, and blue.

Twenty regions of interest (ROIs) were drawn on the CT scan from each session (SHAM and active) by the same investigator to locate the lower limb skeletal muscles. The muscles that comprise the knee extensors (rectus femoris, vastus medialis, vastus intermedius, and vastus lateralis) and knee flexors (semimembranosus, semitendinosus, long head of the biceps femoris, short head of the biceps femoris, gracilis, and sartorius) were identified via visual inspection in the upper leg, and the plantar flexors (gastrocnemius, soleus, peroneus longus, peroneus brevis, flexor digitorum longus, flexor hallucis longus, and tibialis posterior) and dorsiflexors (tibialis anterior, extensor digitorum longus, and extensor hallucis longus) were distinguished in the lower leg. Figure 2 displays a representative upper leg CT image with ROIs identified, a corresponding PET image, and the CT and PET images co-registered. As a result of FDG uptake occurring during the treadmill task, glucose uptake (GU) values closely reflect FDG uptake during the task [18]. For each ROI, standardized uptake values (SUVs) were calculated based on the injected FDG dose and subjects’ body weight. Despite the fasted state of the subjects, SUVs may be affected by varying insulin levels during Sessions 2 and 3 [54,55,56]. Therefore, SUV data were analyzed without normalization and as values normalized to the liver activity as a reference tissue [54,55,56]. Moreover, SUV asymmetry indices (AIs) were calculated to determine the magnitude of asymmetry between the more- and less-affected legs with a previously used equation: ((less-affected side − more-affected side)/((0.5) × (less-affected side + more-affected side)) × 100). An AI value ≥ 10% was considered asymmetric [32,57,58]. The relative distribution ((standard deviation / mean) × 100) of GU values in PET image voxels within each muscular ROI was calculated as an index of spatial glucose uptake heterogeneity (GUh) [27,28]. The data were analyzed using PMOD Version 4.001 (PMD Technologies LLC, Zurich, Switzerland).

### 2.7. Statistical Analysis

Mean ± standard deviation SUVs and GUh for each muscle group (i.e., knee extensors, knee flexors, plantar flexors, and dorsiflexors) were calculated for each subject, and statistical analyses were performed for each muscle group. Normality assumptions were evaluated by way of histograms, Q-Q plots, and the Shapiro–Wilk test. Because these assumptions were met, paired *t*-tests were performed for each participant to compare the muscle groups of each leg between conditions (e.g., left knee extensors during SHAM vs. left knee extensors during tDCS). Significance was accepted at *p* < 0.05, and Cohen’s **d** effect size was calculated for all significant results. Analyses were performed using GraphPad Prism 8.1.2 (GraphPad Software, San Diego, CA, USA).

## 3. Results

All participants completed all of the testing conditions, and no data were missing or removed. Table 1 summarizes the subjects’ characteristics. Data are reported as mean ± SD in the text and tables. The most common sensation reported in the SHAM condition was burning (5.33 ± 2.89), while burning (7.0 ± 3.61) and tingling (5.0 ± 2.83) were most frequently reported in the 3 mA condition. For stimulation blinding, 50% and 75% of subjects correctly guessed the condition after SHAM and 3 mA stimulation, respectively. 

### 3.1. Subject 1

In Subject 1, the peak torque was 127 Nm and 98 Nm for the left and right knee extensors, respectively. Therefore, the anode was placed over the left M1 (C3) to stimulate the area corresponding to the right leg. Treadmill speed was 2.7 mph during both experimental sessions. Blood glucose was 90 mg/dL in Session 1 (SHAM) and 85 mg/dL in Session 2 (tDCS), and the liver SUV mean was 1.96 g/mL and 2.01 g/ML in Sessions 1 and 2, respectively.

#### 3.1.1. Standardized Uptake Values (SUVs)

**Figure 3:**
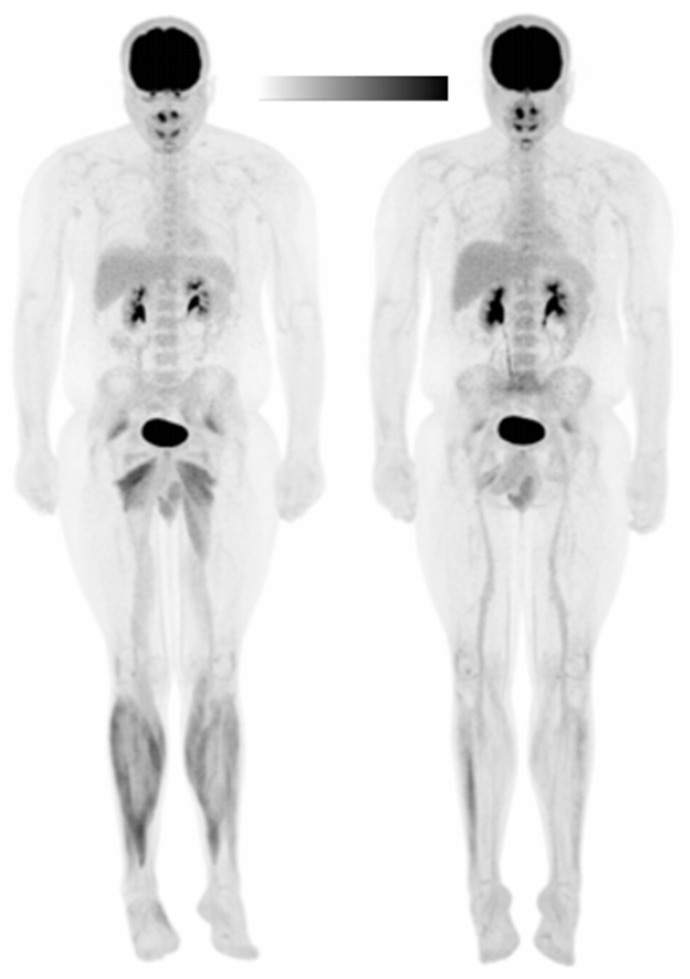
Whole-body maximum intensity projection (MIP) positron emission tomography (PET) image of Subject 1 in the SHAM (**left**) and tDCS (**right**) conditions. A scalar was applied to the tDCS image to normalize to the liver standardized uptake values (SUVs). Black denotes the greatest signal intensity, followed by gray, then white.

Table 2 presents the non-normalized and normalized (i.e., to the liver) glucose uptake standardized uptake values (SUVs) of the leg muscle groups in all four subjects. Supplementary Table S1 shows the SUVs in individual muscles and muscle group averages after SHAM/tDCS and walking in Subject 1 only. SUVs significantly increased after tDCS in the less-affected (left) knee extensors (*p* = 0.008, d = 0.57). Moreover, SUVs significantly decreased after tDCS in the more- and less-affected plantar flexors (more-affected: *p* = 0.002, d = 2.84; less-affected: *p* = 0.007, d = 1.88) and dorsiflexors (more-affected: *p* = 0.005, d = 3.30; less-affected: *p* = 0.016, d = 7.80). Figure 3 provides a representative image of the SUV differences between SHAM (left) and tDCS (right). The muscle groups with significant differences were identical for both normalization values and the *p*- and d-values from the data normalized to the liver are reported.

#### 3.1.2. Asymmetry Indices (AIs)

The SUV AIs after SHAM and tDCS in all four subjects are shown in Table 3. The SUV asymmetry index (AI) went from asymmetrical to symmetrical after tDCS in the plantar flexors and from symmetrical to asymmetrical in the dorsiflexors in Subject 1. No other muscle group had a change in symmetry status after tDCS.

#### 3.1.3. Glucose Uptake Heterogeneity (GUh)

Table 4 presents glucose uptake heterogeneity (GUh) values normalized to the liver in the leg muscle groups of all four subjects. Table S2 presents the GUh of individual muscles as well as muscle group averages in Subject 1 only. GUh significantly decreased in the less-affected plantar flexors (*p* = 0.03, d = 1.27) and dorsiflexors (*p* = 0.01, d = 0.50).

### 3.2. Subject 2

In Subject 2, peak torque was 107 Nm and 145 Nm for the left and right knee extensors, respectively. Therefore, the anode was placed over the right M1 (C4) to stimulate the area corresponding to the left leg. Treadmill speed was 2.9 mph during both experimental sessions. Blood glucose was 95 mg/dL in Session 1 (tDCS) and 98 mg/dL in Session 2 (SHAM) and liver SUV mean was 2.43 g/mL and 2.35 g/ML in Sessions 1 and 2, respectively.

#### 3.2.1. Standardized Uptake Values (SUVs)

Table S3 shows the SUVs in individual muscles and muscle group averages after SHAM/tDCS and walking in Subject 2 only. SUVs significantly increased after tDCS in the more-affected knee flexors (*p* = 0.046, d = 0.40) when non-normalized, but was not significantly different when normalized to the liver (*p* = 0.640). Moreover, SUVs significantly decreased in the less-affected dorsiflexors after tDCS (*p* = 0.045, d = 2.01) both when non-normalized and when normalized to the liver (normalized data are reported).

#### 3.2.2. Asymmetry Indices (AIs)

SUV AIs went from asymmetrical to symmetrical after tDCS in the plantar flexors and dorsiflexors. No other muscle group had a change in symmetry status after tDCS.

#### 3.2.3. Glucose Uptake Heterogeneity (GUh)

Table S4 presents the GUh of individual muscles as well as muscle group averages in Subject 2 only. GUh significantly decreased in the more-affected plantar flexors (*p* = 0.04, d = 0.80) and the less-affected dorsiflexors (*p* = 0.01, d = 6.99).

### 3.3. Subject 3

In Subject 3, peak torque was 59 Nm and 76 Nm for the left and right knee extensors, respectively. Therefore, the anode was placed over the right M1 (C4) to stimulate the area corresponding to the left leg. Treadmill speed was 0.8 mph during both experimental sessions. Blood glucose was 110 mg/dL in Session 1 (SHAM) and 109 mg/dL in Session 2 (tDCS), and the liver SUV mean was 2.41 g/mL and 2.33 g/ML in Sessions 1 and 2, respectively.

#### 3.3.1. Standardized Uptake Values (SUVs)

Table S5 shows the SUVs in individual muscles and muscle group averages after SHAM/tDCS and walking in Subject 3 only. SUVs significantly increased in the less-affected plantar flexors after tDCS (*p* = 0.001, d = 1.33). The muscle groups with significant differences were identical for both normalization values, and the *p*- and d-values from the data normalized to the liver are reported.

#### 3.3.2. Asymmetry Indices (AIs)

SUV AIs went from asymmetrical to symmetrical after tDCS in the plantar flexors and dorsiflexors. No other muscle group had a change in symmetry status after tDCS.

#### 3.3.3. Glucose Uptake Heterogeneity (GUh)

Table S6 presents the GUh of individual muscles as well as muscle group averages in Subject 3 only. GUh significantly decreased in the more- and less-affected knee flexors (more-affected: *p* = 0.03, d = 0.61; less-affected: *p* = 0.02, d = 0.69) and plantar flexors (more-affected: *p* = 0.003, d = 2.24; less-affected: *p* = 0.03, d = 1.54).

### 3.4. Subject 4

In Subject 4, peak torque was 60 Nm and 36 Nm for the left and right knee extensors, respectively. Therefore, the anode was placed over the left M1 (C3) to stimulate the area corresponding to the right leg. Treadmill speed was 3.3 mph during both experimental sessions. Blood glucose was 89 mg/dL in Session 1 (tDCS) and 85 mg/dL in Session 2 (SHAM), the and liver SUV mean was 1.73 g/mL and 1.73 g/ML in Sessions 1 and 2, respectively.

#### 3.4.1. Standardized Uptake Values (SUVs)

Table S7 shows the SUVs in individual muscles and muscle group averages after SHAM/tDCS and walking in Subject 4 only. SUVs significantly decreased in the less-affected knee flexors (*p* = 0.001, d = 0.67) and significantly increased in the less-affected plantar flexors (*p* = 0.035, d = 0.52). The muscle groups with significant differences were identical for both normalization values, and the *p*- and d-values from the data normalized to the liver are reported.

#### 3.4.2. Asymmetry Indices (AIs)

There was no change in SUV symmetry status (e.g., asymmetrical to symmetrical) in any of the muscle groups. However, this subject did not have any muscle groups that exceeded the 10% AI cutoff (Table 3) and was therefore unlikely to experience a symmetry status change.

#### 3.4.3. Glucose Uptake Heterogeneity (GUh)

Table S8 presents the GUh of individual muscles as well as muscle group averages in Subject 4 only. GUh significantly decreased in the less-affected knee flexors (*p* = 0.01, d = 0.46).

## 4. Discussion

The main findings of this study were that a single session of 3 mA tDCS targeting the leg area of the more-affected motor cortex altered glucose uptake (GU) and decreased glucose uptake heterogeneity in various muscle groups in all four PwMS. Moreover, in the three subjects with asymmetrical glucose uptake during SHAM, symmetry status (asymmetric to symmetric) was improved after tDCS. Importantly, the asymmetry index (AI) for the plantar flexors became symmetric after tDCS in these three subjects.

GU increased in the less-affected knee extensors of Subject 1, more-affected knee flexors of Subject 2, and the less-affected plantar flexors of both Subjects 3 and 4. Additionally, GU decreased in the more- and less-affected plantar flexors and dorsiflexors of Subject 1, the less-affected dorsiflexors of Subject 2, and the less-affected knee flexors of Subject 4. This might suggest that tDCS over the motor cortex increases cortical excitability and, therefore, potentially enhances neural drive from the cortex to the spinal motor neuron pool. Importantly, the basic motor pattern for locomotion is generated by central pattern generators (CPGs) [59,60]. However, descending signals from the cortex are required for CPGs to modulate and adapt the rhythmic output appropriately according to the external environment [60,61]. Moreover, distal limb control, which is strongly affected in PwMS, is thought to rely heavily on cortical, cerebellar, and brainstem systems [59,62,63]. Although CPGs cannot be directly affected by tDCS, the cortical excitability of regions that influence CPGs may be altered by stimulation. In the current study, tDCS may have increased excitability of impaired motor regions resulting in improved asymmetries and more effective muscle activation strategies. These data also suggest that some muscles are unaffected or minimally affected by MS pathology, which may explain why glucose uptake in different muscles in each subject was altered by tDCS. For example, similar GU values during both conditions (SHAM and tDCS) may indicate a ceiling effect of tDCS on M1 cortical excitability. In other words, the capacity of motor neuronal stimulation from tDCS to increase MU recruitment, which would alter muscle activation strategies and force production, may be limited in less-impaired muscles compared to muscles with motor impairment. The subjects in this study had a wide age range (27–57 years), time since diagnosis (2–32 years), and varying levels of disability, which may explain discrepancies in which muscles were affected by MS and, therefore, differences in GU. Interestingly, every subject with asymmetrical GU during SHAM (Subjects 1–3) demonstrated improvements in asymmetry in the plantar flexors, which are predominately involved during slow walking [64], after tDCS. These findings are in line with previous studies showing that the muscle groups in the lower limb are most commonly affected in PwMS [2,25,65,66,67] and that plantar flexor weakness, compared to dorsiflexor weakness, is the greater predictor of walking dysfunction in these patients [68]. Therefore, the contribution of lower limb weakness to walking disability in PwMS may partially explain why the largest number of subjects demonstrated improvements in lower leg asymmetries (i.e., in the plantar flexors, followed by the dorsiflexors) in this study.

GUh decreased in the more-affected plantar flexors of Subjects 2 and 3, the more-affected knee flexors of Subject 3, the less-affected plantar flexors of Subjects 1 and 3, the less-affected dorsiflexors of Subjects 1 and 2, and the less-affected knee flexors of Subjects 3 and 4. Additionally, some muscle groups demonstrated decreased GUh independent of changes in GU. For example, Subject 3 decreased GUh in the more-affected plantar flexors as well as the more- and less-affected dorsiflexors despite no significant changes in GU in these muscle groups (Table 2). Moreover, most muscle groups with improvements in AIs (i.e., asymmetric to symmetric; Table 3) also showed significant decreases in GUh (Table 4). The reduction in muscle fibers [31] and the loss of lower motor neurons [69] contribute to the reduced number of motor units within a muscle in PwMS. Fewer motor units may lead to a reduced ability to activate the working muscle homogenously during contractions and could be a contributing factor to strength asymmetries and, therefore, motor deficits and disability in PwMS. In the current study, tDCS over M1 may have effectively increased cortical excitability, resulting in the modulation of motor units. As suggested by animal studies [70], if anodal tDCS intervention increases the recruitment threshold of low-threshold MUs and decreases the recruitment threshold of high threshold MUs, stimulation may facilitate the recruitment of typically difficult-to-recruit MUs and decrease the firing rates of low-threshold MUs. Increased MU activity and subsequent muscle fiber recruitment would result in less GUh, as demonstrated in various muscle groups in the subjects in the current study. These alterations provide evidence that tDCS can have a meaningful effect on peripheral neuromuscular function. However, it is possible that tDCS also increases the recruitment of high threshold MUs, which may exacerbate fatigue in some populations, as previously reported in healthy subjects [50,71].

Interestingly, tDCS affected glucose metabolism in various muscle groups in both the more- and less-affected legs in these subjects. The relatively large size of tDCS electrodes used in this study may explain why effects were observed in both the more- and less-affected legs. These electrodes may have covered other M1 areas in addition to the area that represents the more-affected leg. The use of magnetic resonance imaging (MRI) to guide the localization of scalp positions that correspond to target brain structures [72] or the use of high-definition tDCS (HD-tDCS) in future studies may improve stimulation focality [73]. Furthermore, it has been previously proposed that tDCS of the left hemisphere may influence the right hemisphere [45,74,75]. For example, the results of Mondini et al. [74] demonstrated the effects of tDCS on spectral EEG power on the side contralateral to stimulation, and Park et al. [75] found that stimulation of the left dorsolateral prefrontal cortex had diffuse effects on the right hemisphere areas. The present findings also support the idea that tDCS may affect interhemispheric cooperation of the primary motor cortices during motor performance [76], which may explain, at least in part, the promising findings of this case study.

Only including people with relapsing-remitting MS is a limitation of the study and may limit the generalizability of the results. Moreover, the low temporal resolution of FDG-PET represents another limitation of this study. Specifically, the muscle activation strategies used by the subjects may have shifted from symmetric to asymmetric (or vice versa) during the walking task, and this might not have been detectable with this imaging technique. Moreover, we did not examine how tDCS affects performance outcomes, such as the distance walked during the 6-min walk test, fatigue, or gait characteristics (e.g., step length, gait velocity, time spent in double support). Lastly, 50% and 75% of subjects were able to correctly guess the stimulation condition after SHAM and 3 mA, respectively. Blinding is a common problem in tDCS studies [77,78] and should be addressed in future studies, using alternative SHAM conditions, such as “active SHAM” [79].

Due to the heterogeneity of symptoms, future studies with PwMS should report individual responses to interventions. Moreover, changes in GU, AIs, and GUh should be examined in PwMS with varying levels of disability to determine if this measurement can provide insight into the muscle activity of patients with specific alterations in functionality and/or disability. Examining glucose metabolism may clarify if gait impairments are due to the inability to properly and/or homogenously activate skeletal muscles and can also be used as a tool to monitor the progression of the disease and/or the efficacy of treatments. Moreover, the activity of target brain areas should be assessed pre- and post-tDCS to determine if there is an association between alterations in glucose uptake and/or motor evoked potentials (MEPs) in corticomotor brain areas with changes in leg muscle glucose uptake. Additionally, other variables, such as physical activity level, age, and sex, should be explored and accounted for in these studies. Lastly, the effects of other tDCS parameters, such as multiple sessions, various intensities, and different stimulation sites (e.g., the cerebellum), on glucose metabolism should be assessed.

## 5. Conclusions

tDCS before walking altered glucose uptake and decreased glucose uptake heterogeneity in various muscles in four people with multiple sclerosis. Moreover, glucose uptake became symmetrical after tDCS in the subjects who demonstrated asymmetrical glucose uptake during SHAM. These results suggest that tDCS may have increased cortical excitability and neural drive, resulting in improved muscle activation strategies in these subjects.

## Figures and Tables

**Table 1:** Subject Characteristics.

Subject	Sex	Age (years)	Height (cm)	Weight (kg)	BMI	PDDS	Time Since Diagnosis	Physically Active *
Subject 1	M	27	168	81	28.7	0.0	12 years	No
Subject 2	M	44	183	95	28.4	0.0	2 years	No
Subject 3	M	52	191	111	30.4	3.0	10 years	No
Subject 4	F	57	165	51	18.7	3.0	32 years	Yes

F = Female; M = Male; BMI = Body Mass Index; PDDS = Patient Determined Disease Steps. * Subjects were considered physically active if they self-reported participating in at least 30 min of moderate-intensity physical activity on at least three days of the week for the previous three months.

**Table 2:** Glucose standardized uptake values (SUV in g/mL) in leg muscle groups (knee extensors, knee flexors, plantar flexors, and dorsiflexors) after SHAM/tDCS and walking. The muscle group SUV means ± standard deviations were calculated from the individual muscles that comprise each functional muscle group. Non-normalized (NN) SUVs and SUVs normalized to the liver (NL) are reported.

	NN MA Leg		NN LA Leg		NL MA Leg		NL LA Leg	
	SHAM	tDCS	SHAM	tDCS	SHAM	tDCS	SHAM	tDCS
**Knee Extensors**								
Subject 1	0.48 ± 0.09	0.52 ± 0.08	0.50 ± 0.07	**0.54 ± 0.07 ^±^**	0.25 ± 0.05	0.26 ± 0.04	0.25 ± 0.04	**0.27 ± 0.03 ^±^**
Subject 2	0.59 ± 0.07	0.59 ± 0.06	0.60 ± 0.07	0.59 ± 0.06	0.25 ± 0.03	0.24 ± 0.02	0.25 ± 0.03	0.24 ± 0.02
Subject 3	0.72 ± 0.13	0.61 ± 0.07	0.61 ± 0.05	0.55 ± 0.05	0.30 ± 0.05	0.26 ± 0.03	0.25 ± 0.02	0.24 ± 0.02
Subject 4	0.69 ± 0.06	0.67 ± 0.05	0.69 ± 0.06	0.66 ± 0.04	0.40 ± 0.03	0.39 ± 0.03	0.40 ± 0.03	0.38 ± 0.03
**Knee Flexors**								
Subject 1	0.66 ± 0.28	0.56 ± 0.04	0.63 ± 0.20	0.57 ± 0.03	0.33 ± 0.14	0.28 ± 0.02	0.32 ± 0.10	0.28 ± 0.02
Subject 2	0.64 ± 0.05	**0.66 ± 0.05 ^±^**	0.61 ± 0.05	0.62 ± 0.04	0.27 ± 0.02	0.27 ± 0.02	0.26 ± 0.02	0.26 ± 0.02
Subject 3	0.69 ± 0.08	0.70 ± 0.08	0.62 ± 0.10	0.68 ± 0.21	0.29 ± 0.03	0.30 ± 0.04	0.26 ± 0.04	0.29 ± 0.09
Subject 4	0.66 ± 0.06	0.65 ± 0.06	0.65 ± 0.05	0.63 ± 0.05 *	0.38 ± 0.04	0.38 ± 0.03	0.38 ± 0.03	0.36 ± 0.03 *
**Plantar Flexors**								
Subject 1	2.17 ± 0.60	0.99 ± 0.09 *	1.81 ± 0.60	1.03 ± 0.14 *	1.10 ± 0.30	0.49 ± 0.05 *	0.92 ± 0.30	0.51 ± 0.07 *
Subject 2	1.39 ± 0.82	1.01 ± 0.20	1.12 ± 0.54	0.95 ± 0.19	0.59 ± 0.35	0.42 ± 0.08	0.48 ± 0.23	0.39 ± 0.08
Subject 3	0.96 ± 0.10	0.96 ± 0.07	0.81 ± 0.09	**0.93 ± 0.12 ^±^**	0.40 ± 0.04	0.41 ± 0.03	0.34 ± 0.04	**0.40 ± 0.05 ^±^**
Subject 4	1.00 ± 0.14	1.01 ± 0.14	1.01 ± 0.13	**1.09 ± 0.19 ^±^**	0.58 ± 0.08	0.58 ± 0.08	0.58 ± 0.08	**0.63 ± 0.11 ^±^**
**Dorsiflexors**								
Subject 1	2.39 ± 0.20	1.78 ± 0.21 *	2.25 ± 0.06	1.45 ± 0.14 *	1.21 ± 0.10	0.88 ± 0.10 *	1.14 ± 0.03	0.72 ± 0.07 *
Subject 2	1.01 ± 0.15	1.06 ± 0.13	1.54 ± 0.34	1.05 ± 0.15 *	0.43 ± 0.06	0.44 ± 0.05	0.66 ± 0.15	0.43 ± 0.06 *
Subject 3	1.36 ± 0.08	1.32 ± 0.10	1.06 ± 0.14	1.24 ± 0.26	0.56 ± 0.03	0.57 ± 0.04	0.44 ± 0.06	0.53 ± 0.11
Subject 4	1.10 ± 0.11	1.12 ± 0.14	1.07 ± 0.07	1.08 ± 0.10	0.63 ± 0.06	0.65 ± 0.08	0.62 ± 0.04	0.62 ± 0.06

MA = more-affected; LA = less-affected. * Indicates a significant decrease after tDCS. ^±^ Indicates a significant increase after tDCS. Significance was accepted at *p* < 0.05.

**Table 3:** Standardized uptake value (SUV) asymmetry indices (AIs in %) after SHAM and tDCS.

	Knee Extensors			Knee Flexors			Plantar Flexors			Dorsiflexors		
Subject	SHAM	tDCS	ΔAI	SHAM	tDCS	ΔAI	SHAM	tDCS	ΔAI	SHAM	tDCS	ΔAI
Subject 1	3.23	3.47	−0.24	−4.28	2.21	−6.50	−17.88	**3.58**	−21.46	−6.02	−20.39	14.37
Subject 2	1.08	0.10	0.99	−4.17	−6.45	2.28	−21.22	**−7.00**	−14.22	41.94	**−1.03**	42.97
Subject 3	−16.90	−10.71	−6.19	−10.29	**−3.45**	−6.84	−15.86	**−3.12**	−12.73	−25.21	**−6.76**	−18.45
Subject 4	0.02	−1.11	1.13	−1.00	−3.67	2.66	1.12	7.52	−6.40	−1.99	1.38	−3.37

An AI ≥ 10% was considered asymmetric. Bold indicates a change from asymmetric to symmetric after tDCS.

**Table 4:** Changes in glucose uptake heterogeneity (GUh) for the more- and less-affected muscle groups. The muscle group GUh means ± standard deviations were calculated from the individual muscles that comprise each functional muscle group. A negative number indicates a decrease in GUh in tDCS compared to SHAM.

	MA Leg				LA Leg			
	SHAM	tDCS	ΔGUh	% GUh Change	SHAM	tDCS	ΔGUh	% GUh Change
**Knee Extensors**								
Subject 1	22.54 ± 6.21	21.27 ± 3.41	−1.27 ± 5.91	−1.5 ± 27.46	21.16 ± 6.10	22.37 ± 6.52	1.21 ± 6.33	7.86 ± 28.99
Subject 2	25.81 ± 15.43	22.75 ± 2.47	−3.06 ± 14.00	4.12 ± 35.77	24.86 ± 16.43	23.36 ± 5.74	−1.50 ± 11.33	9.98 ± 33.14
Subject 3	26.02 ± 8.64	21.77 ± 5.61	−4.25 ± 5.96	−14.03 ± 18.01	29.44 ± 14.81	25.35 ± 11.31	−4.09 ± 5.61	−10.39 ± 17.49
Subject 4	19.50 ± 4.65	20.37 ± 4.88	0.87 ± 0.83	4.61 ± 4.97	18.71 ± 4.62	19.01 ± 4.53	0.31 ± 0.77	1.96 ± 4.95
**Knee Flexors**								
Subject 1	23.59 ± 4.59	22.67 ± 7.61	−0.92 ± 8.50	−1.3 ± 33.32	27.40 ± 7.80	22.43 ± 7.88	−4.96 ± 9.53	−13.98 ± 30.54
Subject 2	19.17 ± 2.87	20.22 ± 3.26	1.05 ± 2.66	6.42 ± 16.43	20.25 ± 2.77	21.19 ± 4.53	0.94 ± 2.36	4.04 ± 10.25
Subject 3	22.37 ± 5.75	19.42 ± 3.66 *	−2.95 ± 2.43	−10.43 ± 7.49	24.92 ± 4.21	21.89 ± 4.57 *	−3.02 ± 2.10	−12.32 ± 5.84
Subject 4	17.34 ± 4.72	15.63 ± 3.56	−1.71 ± 2.15	−8.55 ± 13.02	16.97 ± 3.04	15.56 ± 3.12 *	−1.41 ± 0.78	−8.52 ± 6.00
**Plantar Flexors**								
Subject 1	43.32 ± 21.42	28.69 ± 12.63	−14.63 ± 18.85	−25.4 ± 33.22	31.35 ± 6.17	24.34 ± 4.82 *	−7.01 ± 6.71	−20.51 ± 17.47
Subject 2	42.05 ± 15.45	30.72 ± 12.60 *	−11.33 ± 11.21	−26.72 ± 22.81	38.47 ± 9.61	30.19 ± 14.91	−8.28 ± 12.60	−22.58 ± 33.19
Subject 3	35.77 ± 8.41	20.74 ± 4.35 *	−15.03 ± 8.14	−40.16 ± 15.40	34.28 ± 8.74	23.29 ± 5.05 *	−10.99 ± 9.83	−28.63 ± 22.16
Subject 4	21.08 ± 2.66	21.10 ± 3.33	0.02 ± 3.03	0.75 ± 15.07	20.74 ± 3.27	23.08 ± 3.96	2.33 ± 2.62	11.74 ± 12.07
**Dorsiflexors**								
Subject 1	30.78 ± 12.71	33.41 ± 11.06	2.63 ± 6.13	11.30 ± 19.14	27.08 ± 8.46	22.99 ± 7.94 *	−4.09 ± 0.71	−15.72 ± 3.75
Subject 2	23.78 ± 3.08	19.93 ± 3.21	−3.85 ± 4.24	−15.43 ± 17.13	40.29 ± 4.21	18.98 ± 0.93 *	−21.30 ± 4.57	−52.48 ± 6.01
Subject 3	41.63 ± 11.95	24.07 ± 4.74	−17.56 ± 9.97	−40.55 ± 12.16	40.11 ± 18.96	24.87 ± 6.00	−15.24 ± 14.54	−33.32 ± 16.99
Subject 4	19.39 ± 4.30	22.34 ± 8.43	2.95 ± 4.57	13.15 ± 20.94	17.01 ± 2.55	17.15 ± 3.91	0.14 ± 1.74	0.24 ± 9.41

* Indicates a significant decrease in GUh after tDCS. Significance was accepted at *p* < 0.05.

## Data Availability

The data that support the findings of this report will be made available upon request to the corresponding author.

## Supplementary Materials

The following are available online at https://www.mdpi.com/article/10.3390/brainsci11101363/s1, Table S1: Glucose standardized uptake values (SUV in g/mL) in individual muscles and muscle group averages after SHAM/tDCS and walking in Subject 1, Table S2: Changes in glucose uptake heterogeneity (GUh) for the more- and less-affected muscle groups in Subject 1, Table S3: Glucose standardized uptake values (SUV in g/mL) in individual muscles and muscle group averages after SHAM/tDCS and walking in Subject 2, Table S4: Changes in glucose uptake heterogeneity (GUh) for the more- and less-affected muscle groups in Subject 2, Table S5: Glucose standardized uptake values (SUV in g/mL) in individual muscles and muscle group averages after SHAM/tDCS and walking in Subject 3, Table S6: Changes in glucose uptake heterogeneity (GUh) for the more- and less-affected muscle groups in Subject 3, Table S7: Glucose standardized uptake values (SUV in g/mL) in individual muscles and muscle group averages after SHAM/tDCS and walking in Subject 4, and Table S8: Changes in glucose uptake heterogeneity (GUh) for the more- and less-affected muscle groups in Subject 4.
