# Supplementary material for: Alterations in Leg Muscle Glucose Uptake and Inter-Limb Asymmetry after a Single Session of tDCS in Four People with Multiple Sclerosis
Source: Brain Sci. 2021 Oct 16;11(10):1363. doi: 10.3390/brainsci11101363 (PMC8533729; doi:10.3390/brainsci11101363)
Supplement: Supplementary file 1 [file brainsci-11-01363-s001.zip › brainsci-1385350-supplementary.pdf]

Table S1. Glucose standardized uptake values (SUV in g/mL) in individual muscles and muscle group averages after SHAM/tDCS and walking in Subject 1. The right leg was objectively determined to be the more-affected (MA) limb for this subject (LA = less-affected). Non-normalized (NN) SUVs and SUVs normalized to the liver (NL) are reported. Rows in bold represent mean and standard deviation of the respective muscle group.

	NN MA (Right) Leg		NN LA (Left) Leg		NL MA (Right) Leg		NL LA (Left) Leg	
**Muscle**	**SHAM**	**tDCS**	**SHAM**	**tDCS**	**SHAM**	**tDCS**	**SHAM**	**tDCS**
**Knee Extensors**								
Rectus Femoris	0.37 ± 0.12	0.44 ± 0.10	0.42 ± 0.13	0.46 ± 0.11	0.19 ± 0.06	0.22 ± 0.05	0.21 ± 0.06	0.23 ± 0.06
Vastus Medialis	0.49 ± 0.11	0.49 ± 0.11	0.49 ± 0.10	0.54 ± 0.16	0.25 ± 0.06	0.25 ± 0.05	0.25 ± 0.05	0.27 ± 0.08
Vastus Intermedius	0.60 ± 0.10	0.62 ± 0.15	0.59 ± 0.11	0.62 ± 0.12	0.30 ± 0.05	0.31 ± 0.07	0.30 ± 0.05	0.31 ± 0.06
Vastus Lateralis	0.48 ± 0.09	0.52 ± 0.09	0.49 ± 0.08	0.53 ± 0.08	0.24 ± 0.05	0.26 ± 0.04	0.25 ± 0.04	0.27 ± 0.04
**Mean ± SD**	**0.48 ± 0.09**	**0.52 ± 0.08**	**0.50 ± 0.07**	**0.54 ± 0.07±**	**0.25 ± 0.05**	**0.26 ± 0.04**	**0.25 ± 0.04**	**0.27 ± 0.03±**
**Knee Flexors**								
BF – Long Head	0.56 ± 0.10	0.53 ± 0.12	0.56 ± 0.11	0.54 ± 0.12	0.29 ± 0.05	0.26 ± 0.06	0.29 ± 0.06	0.27 ± 0.06
BF – Short Head	0.55 ± 0.10	0.61 ± 0.13	0.57 ± 0.09	0.61 ± 0.09	0.28 ± 0.05	0.30 ± 0.06	0.29 ± 0.05	0.30 ± 0.05
Semimembranosus	1.23 ± 0.29	0.58 ± 0.09	1.03 ± 0.32	0.60 ± 0.10	0.62 ± 0.05	0.29 ± 0.05	0.52 ± 0.16	0.30 ± 0.05
Semitendinosus	0.55 ± 0.16	0.54 ± 0.10	0.57 ± 0.19	0.57 ± 0.10	0.28 ± 0.08	0.27 ± 0.05	0.29 ± 0.10	0.29 ± 0.05
Sartorius	0.48 ± 0.13	0.51 ± 0.19	0.43 ± 0.13	0.52 ± 0.19	0.25 ± 0.06	0.25 ± 0.10	0.22 ± 0.06	0.26 ± 0.10
Gracilis	0.57 ± 0.15	0.56 ± 0.11	0.62 ± 0.22	0.56 ± 0.14	0.29 ± 0.07	0.28 ± 0.06	0.32 ± 0.11	0.28 ± 0.07
**Mean ± SD**	**0.66 ± 0.28**	**0.56 ± 0.04**	**0.63 ± 0.20**	**0.57 ± 0.03**	**0.33 ± 0.14**	**0.28 ± 0.02**	**0.32 ± 0.10**	**0.28 ± 0.02**
**Plantar Flexors**								
Gastrocnemius	2.18 ± 0.86	0.95 ± 0.21	2.11 ± 0.74	1.00 ± 0.27	1.11 ± 0.44	0.47 ± 0.10	1.08 ± 0.38	0.50 ± 0.13
Soleus	2.62 ± 0.60	0.95 ± 0.14	2.00 ± 0.57	1.00 ± 0.18	1.34 ± 0.30	0.47 ± 0.07	1.02 ± 0.29	0.50 ± 0.09
Peroneus Longus	1.50 ± 1.18	0.84 ± 0.27	0.97 ± 0.24	0.81 ± 0.17	0.77 ± 0.60	0.42 ± 0.13	0.49 ± 0.12	0.40 ± 0.08
Peroneus Brevis	1.45 ± 0.89	1.08 ± 0.59	1.15 ± 0.41	0.94 ± 0.29	0.74 ± 0.45	0.54 ± 0.29	0.58 ± 0.21	0.47 ± 0.14
Flexor Digitorum	3.02 ± 1.56	0.94 ± 0.19	2.72 ± 1.09	1.08 ± 0.22	1.54 ± 0.79	0.47 ± 0.10	1.38 ± 0.56	0.54 ± 0.11
Flexor Hallucis	2.56 ± 0.66	1.08 ± 0.31	2.00 ± 0.65	1.13 ± 0.33	1.30 ± 0.34	0.47 ± 0.10	1.02 ± 0.33	0.56 ± 0.17
Tibialis Posterior	1.86 ± 0.44	1.09 ± 0.31	1.76 ± 0.41	1.22 ± 0.29	0.95 ± 0.23	0.54 ± 0.16	0.89 ± 0.21	0.61 ± 0.15
**Mean ± SD**	**2.17 ± 0.60**	**0.99 ± 0.09***	**1.81 ± 0.60**	**1.03 ± 0.14***	**1.10 ± 0.30**	**0.49 ± 0.05***	**0.92 ± 0.30**	**0.51 ± 0.07***
**Dorsiflexors**								
Tibialis Anterior	2.22 ± 0.42	1.56 ± 0.32	2.28 ± 0.46	1.28 ± 0.21	1.13 ± 0.21	0.77 ± 0.16	1.16 ± 0.23	0.64 ± 0.10
Extensor Digitorum	2.32 ± 1.03	1.80 ± 0.74	2.18 ± 0.80	1.56 ± 0.49	1.18 ± 0.52	0.90 ± 0.37	1.11 ± 0.41	0.78 ± 0.25
Extensor Hallucis	2.61 ± 0.76	1.97 ± 0.76	2.28 ± 0.56	1.50 ± 0.32	1.33 ± 0.39	0.98 ± 0.38	1.16 ± 0.28	0.75 ± 0.16
**Mean ± SD**	**2.39 ± 0.20**	**1.78 ± 0.21***	**2.25 ± 0.06**	**1.45 ± 0.14***	**1.21 ± 0.10**	**0.88 ± 0.10***	**1.14 ± 0.03**	**0.72 ± 0.07***

*****Indicates significant decrease after tDCS. ±Indicates significant increase after tDCS. Significance was accepted at $p < 0.05$.

Table S2. Changes in glucose uptake heterogeneity (GUh) for the more- and less-affected muscle groups in Subject 1. Rows in bold represent mean and standard deviation of the respective muscle group. A negative number indicates a decrease in GUh in tDCS compared to SHAM.

Muscle	MA (Right) Leg				LA (Left) Leg			
	SHAM	tDCS	ΔGUh	% GUh Change	SHAM	tDCS	ΔGUh	% GUh Change
**Knee Extensors**								
Rectus Femoris	31.15	23.39	-7.76	-24.92	29.98	24.87	-5.11	-17.05
Vastus Medialis	22.91	21.47	-1.44	-6.29	20.25	30.04	9.79	48.37
Vastus Intermedius	17.27	23.83	6.56	37.99	18.09	19.60	1.51	8.36
Vastus Lateralis	18.83	16.40	-2.44	-12.94	16.32	14.98	-1.35	-8.25
**Mean ± SD**	**22.54 ± 6.21**	**21.27 ± 3.41**	**-1.27 ± 5.91**	**-1.5 ± 27.46**	**21.16 ± 6.10**	**22.37 ± 6.52**	**1.21 ± 6.33**	**7.86 ± 28.99**
**Knee Flexors**								
BF – Long Head	18.24	22.51	4.27	23.39	19.53	22.12	2.59	13.28
BF – Short Head	18.20	21.17	2.97	16.33	16.02	15.32	-0.70	-4.40
Semimembranosus	23.69	16.27	-7.43	-31.35	31.16	17.07	-14.09	-45.21
Semitendinosus	29.66	18.50	-11.16	-37.62	33.84	18.05	-15.79	-46.66
Sartorius	26.20	37.56	11.36	43.36	29.08	36.70	7.61	26.18
Gracilis	25.57	20.03	-5.54	-21.67	34.77	25.35	-9.41	-27.08
**Mean ± SD**	**23.59 ± 4.59**	**22.67 ± 7.61**	**-0.92 ± 8.50**	**-1.3 ± 33.32**	**27.40 ± 7.80**	**22.43 ± 7.88**	**-4.96 ± 9.53**	**-13.98 ± 30.54**
**Plantar Flexors**								
Gastrocnemius	39.43	22.20	-17.23	-43.71	34.95	26.70	-8.25	-23.60
Soleus	22.79	14.59	-8.20	-35.96	28.49	18.46	-10.03	-35.20
Peroneus Longus	78.59	31.50	-47.09	-59.92	24.61	20.77	-3.84	-15.61
Peroneus Brevis	61.22	54.01	-7.21	-11.77	35.52	30.86	-4.66	-13.13
Flexor Digitorum	51.56	20.55	-31.00	-60.14	40.20	20.20	-20.01	-49.77
Flexor Hallucis	25.77	29.23	3.46	13.42	32.38	29.51	-2.87	-8.87
Tibialis Posterior	23.91	28.75	4.84	20.24	23.28	23.89	0.61	2.63
**Mean ± SD**	**43.32 ± 21.42**	**28.69 ± 12.63**	**-14.63 ± 18.85**	**-25.4 ± 33.22**	**31.35 ± 6.17**	**24.34 ± 4.82***	**-7.01 ± 6.71**	**-20.51 ± 17.47**
**Dorsiflexors**								
Tibialis Anterior	18.93	20.75	1.82	9.62	20.16	16.12	-4.04	-20.03
Extensor Digitorum	44.20	41.15	-3.05	-6.91	36.51	31.68	-4.83	-13.23
Extensor Hallucis	29.21	38.34	9.13	31.26	24.57	21.15	-3.41	-13.90
**Mean ± SD**	**30.78 ± 12.71**	**33.41 ± 11.06**	**2.63 ± 6.13**	**11.30 ± 19.14**	**27.08 ± 8.46**	**22.99 ± 7.94***	**-4.09 ± 0.71**	**-15.72 ± 3.75**

*Indicates significant decrease in GUh after tDCS. Significance was accepted at $p < 0.05$.

Table S3. Glucose standardized uptake values (SUV in g/mL) in individual muscles and muscle group averages after SHAM/tDCS and walking in Subject 2. The left leg was objectively determined to be the more-affected (MA) limb for this subject (LA = less-affected). Non-normalized (NN) SUVs and SUVs normalized to the liver (NL) are reported. Rows in bold represent mean and standard deviation of the respective muscle group.

	NN MA (Left) Leg		NN LA (Right) Leg		NL MA (Left) Leg		NL LA (Right) Leg	
**Muscle**	**SHAM**	**tDCS**	**SHAM**	**tDCS**	**SHAM**	**tDCS**	**SHAM**	**tDCS**
**Knee Extensors**								
Rectus Femoris	0.50 ± 0.09	0.53 ± 0.12	0.51 ± 0.08	0.54 ± 0.10	0.22 ± 0.04	0.22 ± 0.05	0.22 ± 0.04	0.22 ± 0.04
Vastus Medialis	0.64 ± 0.31	0.57 ± 0.14	0.67 ± 0.33	0.59 ± 0.18	0.27 ± 0.13	0.24 ± 0.06	0.29 ± 0.14	0.24 ± 0.07
Vastus Intermedius	0.64 ± 0.13	0.67 ± 0.17	0.63 ± 0.13	0.67 ± 0.17	0.27 ± 0.06	0.28 ± 0.07	0.27 ± 0.06	0.27 ± 0.07
Vastus Lateralis	0.57 ± 0.09	0.57 ± 0.11	0.57 ± 0.09	0.55 ± 0.10	0.24 ± 0.04	0.23 ± 0.05	0.24 ± 0.04	0.23 ± 0.04
**Mean ± SD**	**0.59 ± 0.07**	**0.59 ± 0.06**	**0.60 ± 0.07**	**0.59 ± 0.06**	**0.25 ± 0.03**	**0.24 ± 0.02**	**0.25 ± 0.03**	**0.24 ± 0.02**
**Knee Flexors**								
BF – Long Head	0.64 ± 0.13	0.65 ± 0.13	0.59 ± 0.13	0.60 ± 0.14	0.27 ± 0.05	0.27 ± 0.05	0.25 ± 0.06	0.25 ± 0.06
BF – Short Head	0.72 ± 0.14	0.76 ± 0.13	0.69 ± 0.14	0.69 ± 0.13	0.31 ± 0.06	0.31 ± 0.06	0.29 ± 0.06	0.28 ± 0.05
Semimembranosus	0.66 ± 0.10	0.68 ± 0.14	0.62 ± 0.10	0.64 ± 0.11	0.28 ± 0.04	0.28 ± 0.06	0.27 ± 0.04	0.27 ± 0.04
Semitendinosus	0.57 ± 0.10	0.63 ± 0.11	0.56 ± 0.10	0.61 ± 0.11	0.25 ± 0.04	0.26 ± 0.05	0.24 ± 0.04	0.25 ± 0.05
Sartorius	0.63 ± 0.15	0.64 ± 0.17	0.63 ± 0.15	0.62 ± 0.18	0.27 ± 0.06	0.26 ± 0.07	0.27 ± 0.06	0.26 ± 0.07
Gracilis	0.59 ± 0.11	0.61 ± 0.13	0.56 ± 0.11	0.58 ± 0.12	0.25 ± 0.05	0.25 ± 0.05	0.24 ± 0.05	0.24 ± 0.05
**Mean ± SD**	**0.64 ± 0.05**	**0.66 ± 0.05**±	**0.61 ± 0.05**	**0.62 ± 0.04**	**0.27 ± 0.02**	**0.27 ± 0.02**	**0.26 ± 0.02**	**0.26 ± 0.02**
**Plantar Flexors**								
Gastrocnemius	1.06 ± 0.45	1.09 ± 0.46	0.83 ± 0.26	0.86 ± 0.24	0.45 ± 0.19	0.45 ± 0.19	0.36 ± 0.11	0.36 ± 0.10
Soleus	1.43 ± 0.83	1.03 ± 0.37	1.11 ± 0.55	0.88 ± 0.30	0.61 ± 0.35	0.42 ± 0.15	0.47 ± 0.23	0.36 ± 0.12
Peroneus Longus	0.72 ± 0.22	0.81 ± 0.12	0.72 ± 0.23	0.76 ± 0.11	0.31 ± 0.09	0.33 ± 0.05	0.31 ± 0.10	0.31 ± 0.04
Peroneus Brevis	0.75 ± 0.16	0.82 ± 0.10	0.83 ± 0.23	0.85 ± 0.11	0.32 ± 0.07	0.34 ± 0.04	0.36 ± 0.10	0.35 ± 0.05
Flexor Digitorum	1.57 ± 0.51	1.10 ± 0.34	0.97 ± 0.35	0.96 ± 0.28	0.67 ± 0.22	0.45 ± 0.14	0.41 ± 0.15	0.39 ± 0.12
Flexor Hallucis	3.11 ± 1.39	1.36 ± 0.59	2.31 ± 0.93	1.36 ± 0.78	1.32 ± 0.59	0.56 ± 0.24	0.98 ± 0.40	0.56 ± 0.32
Tibialis Posterior	1.09 ± 0.71	0.90 ± 0.33	1.08 ± 0.57	0.95 ± 0.33	0.47 ± 0.30	0.37 ± 0.14	0.46 ± 0.24	0.39 ± 0.14
**Mean ± SD**	**1.39 ± 0.82**	**1.01 ± 0.20**	**1.12 ± 0.54**	**0.95 ± 0.19**	**0.59 ± 0.35**	**0.42 ± 0.08**	**0.48 ± 0.23**	**0.39 ± 0.08**
**Dorsiflexors**								
Tibialis Anterior	1.17 ± 0.32	1.18 ± 0.24	1.88 ± 0.67	1.18 ± 0.22	0.50 ± 0.14	0.48 ± 0.10	0.80 ± 0.29	0.49 ± 0.09
Extensor Digitorum	0.89 ± 0.20	0.92 ± 0.21	1.19 ± 0.49	0.88 ± 0.18	0.38 ± 0.08	0.38 ± 0.09	0.51 ± 0.21	0.36 ± 0.07
Extensor Hallucis	0.96 ± 0.21	1.09 ± 0.18	1.56 ± 0.68	1.09 ± 0.20	0.41 ± 0.09	0.45 ± 0.07	0.66 ± 0.29	0.45 ± 0.08
**Mean ± SD**	**1.01 ± 0.15**	**1.06 ± 0.13**	**1.54 ± 0.34**	**1.05 ± 0.15***	**0.43 ± 0.06**	**0.44 ± 0.05**	**0.66 ± 0.15**	**0.43 ± 0.06***

***I**ndicates significant decrease after tDCS. ±Indicates significant increase after tDCS. Significance was accepted at $p < 0.05$.

Table S4. Changes in glucose uptake heterogeneity (GUh) for the more- and less-affected muscle groups in Subject 2. Rows in bold represent mean and standard deviation of the respective muscle group. A negative number indicates a decrease in GUh in tDCS compared to SHAM.

	MA (Left) Leg				LA (Right) Leg			
**Muscle**	**SHAM**	**tDCS**	**ΔGUh**	**% GUh Change**	**SHAM**	**tDCS**	**ΔGUh**	**% GUh Change**
**Knee Extensors**								
Rectus Femoris	18.38	21.99	3.61	19.64	13.37	18.63	5.26	39.36
Vastus Medialis	48.75	24.69	-24.06	-49.36	49.08	30.66	-18.41	-37.52
Vastus Intermedius	20.56	24.73	4.17	20.29	20.78	25.22	4.44	21.35
Vastus Lateralis	15.55	19.58	4.03	25.92	16.23	18.94	2.71	16.71
**Mean ± SD**	**25.81 ± 15.43**	**22.75 ± 2.47**	**-3.06 ± 14.00**	**4.12 ± 35.77**	**24.86 ± 16.43**	**23.36 ± 5.74**	**-1.50 ± 11.33**	**9.98 ± 33.14**
**Knee Flexors**								
BF – Long Head	20.03	19.18	-0.85	-4.24	22.54	22.86	0.32	1.42
BF – Short Head	19.42	17.62	-1.79	-9.25	20.52	18.65	-1.87	-9.12
Semimembranosus	14.86	20.23	5.37	36.14	16.70	16.87	0.18	1.06
Semitendinosus	17.96	17.49	-0.47	-2.63	17.95	18.02	0.07	0.41
Sartorius	23.67	26.38	2.71	11.44	24.10	29.22	5.12	21.25
Gracilis	19.11	20.45	1.35	7.04	19.71	21.53	1.81	9.20
**Mean ± SD**	**19.17 ± 2.87**	**20.22 ± 3.26**	**1.05 ± 2.66**	**6.42 ± 16.43**	**20.25 ± 2.77**	**21.19 ± 4.53**	**0.94 ± 2.36**	**4.04 ± 10.25**
**Plantar Flexors**								
Gastrocnemius	42.18	42.18	0.00	0.00	30.58	27.97	-2.61	-8.53
Soleus	57.78	35.66	-22.12	-38.28	49.13	33.78	-15.36	-31.25
Peroneus Longus	44.75	43.05	-1.70	-3.81	31.53	14.07	-17.46	-55.38
Peroneus Brevis	21.70	11.91	-9.78	-45.09	28.05	13.36	-14.69	-52.36
Flexor Digitorum	32.35	30.93	-1.42	-4.40	36.49	29.63	-6.86	-18.79
Flexor Hallucis	44.75	43.05	-1.70	-3.81	40.43	57.59	17.15	42.42
Tibialis Posterior	65.16	36.60	-28.56	-43.82	53.07	34.95	-18.12	-34.14
**Mean ± SD**	**42.05 ± 15.45**	**30.72 ± 12.60***	**-11.33 ± 11.21**	**-26.72 ± 22.81**	**38.47 ± 9.61**	**30.19 ± 14.91**	**-8.28 ± 12.60**	**-22.58 ± 33.19**
**Dorsiflexors**								
Tibialis Anterior	27.34	20.28	-7.06	-25.82	35.72	18.92	-16.79	-47.02
Extensor Digitorum	22.00	22.95	0.96	4.34	41.13	19.94	-21.19	-51.51
Extensor Hallucis	22.01	16.55	-5.46	-24.81	44.01	18.08	-25.93	-58.92
**Mean ± SD**	**23.78 ± 3.08**	**19.93 ± 3.21**	**-3.85 ± 4.24**	**-15.43 ± 17.13**	**40.29 ± 4.21**	**18.98 ± 0.93***	**-21.30 ± 4.57**	**-52.48 ± 6.01**

*Indicates significant decrease in GUh after tDCS. Significance was accepted at $p < 0.05$.

Table S5. Glucose standardized uptake values (SUV in g/mL) in individual muscles and muscle group averages after SHAM/tDCS and walking in Subject 3. The left leg was objectively determined to be the more-affected (MA) limb for this subject (LA = less-affected). Non-normalized (NN) SUVs and SUVs normalized to the liver (NL) are reported. Rows in bold represent mean and standard deviation of the respective muscle group.

	**NN MA (Left) Leg**		**NN LA (Right) Leg**		**NL MA (Left) Leg**		**NL LA (Right) Leg**	
**Muscle**	**SHAM**	**tDCS**	**SHAM**	**tDCS**	**SHAM**	**tDCS**	**SHAM**	**tDCS**
**Knee Extensors**								
Rectus Femoris	0.55 ± 0.13	0.55 ± 0.14	0.55 ± 0.11	0.51 ± 0.11	0.23 ± 0.05	0.23 ± 0.06	0.23 ± 0.04	0.22 ± 0.05
Vastus Medialis	0.79 ± 0.30	0.57 ± 0.15	0.66 ± 0.32	0.54 ± 0.22	0.33 ± 0.12	0.25 ± 0.06	0.27 ± 0.13	0.23 ± 0.10
Vastus Intermedius	0.84 ± 0.22	0.71 ± 0.15	0.65 ± 0.21	0.62 ± 0.14	0.35 ± 0.09	0.31 ± 0.06	0.27 ± 0.09	0.27 ± 0.06
Vastus Lateralis	0.69 ± 0.12	0.63 ± 0.09	0.57 ± 0.09	0.54 ± 0.08	0.28 ± 0.05	0.27 ± 0.04	0.24 ± 0.04	0.23 ± 0.03
**Mean ± SD**	**0.72 ± 0.13**	**0.61 ± 0.07**	**0.61 ± 0.05**	**0.55 ± 0.05**	**0.30 ± 0.05**	**0.26 ± 0.03**	**0.25 ± 0.02**	**0.24 ± 0.02**
**Knee Flexors**								
BF – Long Head	0.59 ± 0.14	0.57 ± 0.12	0.50 ± 0.12	0.51 ± 0.09	0.24 ± 0.06	0.24 ± 0.05	0.21 ± 0.05	0.22 ± 0.04
BF – Short Head	0.78 ± 0.14	0.73 ± 0.12	0.70 ± 0.16	0.65 ± 0.12	0.32 ± 0.06	0.31 ± 0.05	0.29 ± 0.07	0.28 ± 0.05
Semimembranosus	0.73 ± 0.13	0.74 ± 0.13	0.62 ± 0.14	0.64 ± 0.13	0.30 ± 0.05	0.32 ± 0.06	0.26 ± 0.06	0.28 ± 0.05
Semitendinosus	0.64 ± 0.13	0.66 ± 0.12	0.53 ± 0.11	0.57 ± 0.11	0.27 ± 0.05	0.28 ± 0.05	0.22 ± 0.05	0.24 ± 0.05
Sartorius	0.76 ± 0.25	0.82 ± 0.21	0.60 ± 0.19	0.63 ± 0.18	0.32 ± 0.10	0.35 ± 0.09	0.25 ± 0.08	0.27 ± 0.08
Gracilis	0.63 ± 0.13	0.71 ± 0.12	0.78 ± 0.21	1.09 ± 0.30	0.26 ± 0.05	0.30 ± 0.05	0.32 ± 0.09	0.47 ± 0.13
**Mean ± SD**	**0.69 ± 0.08**	**0.70 ± 0.08**	**0.62 ± 0.10**	**0.68 ± 0.21**	**0.29 ± 0.03**	**0.30 ± 0.04**	**0.26 ± 0.04**	**0.29 ± 0.09**
**Plantar Flexors**								
Gastrocnemius	0.97 ± 0.41	0.98 ± 0.25	0.69 ± 0.29	0.86 ± 0.17	0.40 ± 0.17	0.42 ± 0.11	0.29 ± 0.12	0.37 ± 0.07
Soleus	1.01 ± 0.27	0.93 ± 0.20	0.80 ± 0.22	0.88 ± 0.23	0.42 ± 0.11	0.40 ± 0.09	0.33 ± 0.09	0.38 ± 0.10
Peroneus Longus	0.80 ± 0.29	0.94 ± 0.12	0.78 ± 0.27	0.77 ± 0.13	0.33 ± 0.12	0.40 ± 0.05	0.33 ± 0.11	0.33 ± 0.06
Peroneus Brevis	0.92 ± 0.27	1.10 ± 0.20	0.72 ± 0.22	0.85 ± 0.18	0.38 ± 0.11	0.47 ± 0.09	0.30 ± 0.09	0.36 ± 0.08
Flexor Digitorum	1.06 ± 0.35	0.87 ± 0.21	0.92 ± 0.22	1.07 ± 0.22	0.44 ± 0.15	0.37 ± 0.09	0.38 ± 0.09	0.46 ± 0.09
Flexor Hallucis	1.06 ± 0.33	0.96 ± 0.18	0.88 ± 0.28	1.02 ± 0.32	0.44 ± 0.14	0.41 ± 0.08	0.36 ± 0.12	0.44 ± 0.14
Tibialis Posterior	0.88 ± 0.45	0.93 ± 0.22	0.92 ± 0.46	1.05 ± 0.27	0.36 ± 0.19	0.40 ± 0.09	0.38 ± 0.19	0.45 ± 0.12
**Mean ± SD**	**0.96 ± 0.10**	**0.96 ± 0.07**	**0.81 ± 0.09**	**0.93 ± 0.12**[±]	**0.40 ± 0.04**	**0.41 ± 0.03**	**0.34 ± 0.04**	**0.40 ± 0.05**[±]
**Dorsiflexors**								
Tibialis Anterior	1.27 ± 0.69	1.43 ± 0.36	1.06 ± 0.65	1.52 ± 0.45	0.53 ± 0.29	0.61 ± 0.16	0.44 ± 0.27	0.65 ± 0.19
Extensor Digitorum	1.42 ± 0.56	1.22 ± 0.34	0.92 ± 0.30	1.03 ± 0.28	0.59 ± 0.23	0.52 ± 0.15	0.38 ± 0.13	0.44 ± 0.12
Extensor Hallucis	1.39 ± 0.43	1.32 ± 0.25	1.20 ± 0.31	1.16 ± 0.21	0.58 ± 0.18	0.57 ± 0.11	0.50 ± 0.13	0.50 ± 0.09
**Mean ± SD**	**1.36 ± 0.08**	**1.32 ± 0.10**	**1.06 ± 0.14**	**1.24 ± 0.26**	**0.56 ± 0.03**	**0.57 ± 0.04**	**0.44 ± 0.06**	**0.53 ± 0.11**

*Indicates significant decrease after tDCS. [±]Indicates significant increase after tDCS. Significance was accepted at $p < 0.05$.

Table S6. Changes in glucose uptake heterogeneity (GUh) for the more- and less-affected muscle groups in Subject 3. Rows in bold represent mean and standard deviation of the respective muscle group. A negative number indicates a decrease in GUh in tDCS compared to SHAM.

	MA (Left) Leg				LA (Right) Leg			
**Muscle**	**SHAM**	**tDCS**	**ΔGUh**	**% GUh Change**	**SHAM**	**tDCS**	**ΔGUh**	**% GUh Change**
**Knee Extensors**								
Rectus Femoris	23.46	26.12	2.66	11.35	19.60	21.92	2.32	11.83
Vastus Medialis	37.84	26.07	-11.77	-31.10	48.90	41.48	-7.41	-15.16
Vastus Intermedius	25.55	20.59	-4.95	-19.39	32.89	22.94	-9.95	-30.27
Vastus Lateralis	17.23	14.30	-2.93	-16.98	16.36	15.06	-1.30	-7.96
**Mean ± SD**	**26.02 ± 8.64**	**21.77 ± 5.61**	**-4.25 ± 5.96**	**-14.03 ± 18.01**	**29.44 ± 14.81**	**25.35 ± 11.31**	**-4.09 ± 5.61**	**-10.39 ± 17.49**
**Knee Flexors**								
BF – Long Head	24.61	21.32	-3.29	-13.37	24.40	18.45	-5.95	-24.38
BF – Short Head	18.11	16.59	-1.52	-8.38	22.72	19.01	-3.72	-16.37
Semimembranosus	18.11	17.51	-0.60	-3.31	22.45	19.69	-2.76	-12.29
Semitendinosus	19.69	18.58	-1.11	-5.63	20.59	18.70	-1.89	-9.20
Sartorius	33.05	25.96	-7.09	-21.45	32.27	28.20	-4.07	-12.60
Gracilis	20.66	16.58	-4.08	-19.75	27.06	27.31	0.24	0.89
**Mean ± SD**	**22.37 ± 5.75**	**19.42 ± 3.66***	**-2.95 ± 2.43**	**-10.43 ± 7.49**	**24.92 ± 4.21**	**21.89 ± 4.57***	**-3.02 ± 2.10**	**-12.32 ± 5.84**
**Plantar Flexors**								
Gastrocnemius	42.32	25.28	-17.04	-40.27	41.46	19.88	-21.59	-52.06
Soleus	27.27	21.79	-5.48	-20.09	27.46	26.34	-1.11	-4.05
Peroneus Longus	35.96	12.75	-23.21	-64.54	34.24	17.15	-17.09	-49.92
Peroneus Brevis	29.62	18.59	-11.03	-37.24	31.29	21.53	-9.76	-31.20
Flexor Digitorum	32.96	24.48	-8.49	-25.75	23.85	20.20	-3.65	-15.30
Flexor Hallucis	30.96	18.99	-11.97	-38.68	31.99	31.87	-0.12	-0.37
Tibialis Posterior	51.28	23.31	-27.97	-54.54	49.69	26.08	-23.61	-47.51
**Mean ± SD**	**35.77 ± 8.41**	**20.74 ± 4.35***	**-15.03 ± 8.14**	**-40.16 ± 15.40**	**34.28 ± 8.74**	**23.29 ± 5.05***	**-10.99 ± 9.83**	**-28.63 ± 22.16**
**Dorsiflexors**								
Tibialis Anterior	54.53	25.47	-29.07	-53.30	61.54	29.52	-32.02	-52.03
Extensor Digitorum	39.42	27.96	-11.46	-29.07	33.26	26.99	-6.27	-18.85
Extensor Hallucis	30.94	18.78	-12.15	-39.29	25.52	18.10	-7.42	-29.07
**Mean ± SD**	**41.63 ± 11.95**	**24.07 ± 4.74**	**-17.56 ± 9.97**	**-40.55 ± 12.16**	**40.11 ± 18.96**	**24.87 ± 6.00**	**-15.24 ± 14.54**	**-33.32 ± 16.99**

*Indicates significant decrease in GUh after tDCS. Significance was accepted at $p < 0.05$.

Table S7. Glucose standardized uptake values (SUV in g/mL) in individual muscles and muscle group averages after SHAM/tDCS and walking in Subject 4. The right leg was objectively determined to be the more-affected (MA) limb for this subject (LA = less-affected). Non-normalized (NN) SUVs and SUVs normalized to the liver (NL) are reported. Rows in bold represent mean and standard deviation of the respective muscle group.

	**NN MA (Right) Leg**		**NN LA (Left) Leg**		**NL MA (Right) Leg**		**NL LA (Left) Leg**	
**Muscle**	**SHAM**	**tDCS**	**SHAM**	**tDCS**	**SHAM**	**tDCS**	**SHAM**	**tDCS**
**Knee Extensors**								
Rectus Femoris	0.70 ± 0.17	0.67 ± 0.17	0.67 ± 0.15	0.68 ± 0.15	0.40 ± 0.10	0.39 ± 0.10	0.39 ± 0.09	0.39 ± 0.08
Vastus Medialis	0.68 ± 0.15	0.64 ± 0.15	0.66 ± 0.15	0.64 ± 0.15	0.39 ± 0.09	0.37 ± 0.09	0.38 ± 0.09	0.37 ± 0.09
Vastus Intermedius	0.76 ± 0.11	0.74 ± 0.11	0.78 ± 0.11	0.72 ± 0.11	0.44 ± 0.06	0.43 ± 0.07	0.45 ± 0.06	0.42 ± 0.06
Vastus Lateralis	0.63 ± 0.11	0.64 ± 0.11	0.65 ± 0.10	0.62 ± 0.10	0.36 ± 0.06	0.37 ± 0.06	0.38 ± 0.06	0.36 ± 0.06
**Mean ± SD**	**0.69 ± 0.06**	**0.67 ± 0.05**	**0.69 ± 0.06**	**0.66 ± 0.04**	**0.40 ± 0.03**	**0.39 ± 0.03**	**0.40 ± 0.03**	**0.38 ± 0.03**
**Knee Flexors**								
BF – Long Head	0.60 ± 0.11	0.60 ± 0.11	0.60 ± 0.11	0.57 ± 0.10	0.34 ± 0.06	0.35 ± 0.06	0.35 ± 0.06	0.33 ± 0.06
BF – Short Head	0.74 ± 0.09	0.73 ± 0.10	0.74 ± 0.10	0.71 ± 0.08	0.43 ± 0.05	0.42 ± 0.06	0.42 ± 0.06	0.41 ± 0.05
Semimembranosus	0.62 ± 0.09	0.60 ± 0.07	0.66 ± 0.09	0.64 ± 0.09	0.36 ± 0.05	0.34 ± 0.04	0.38 ± 0.05	0.37 ± 0.05
Semitendinosus	0.63 ± 0.10	0.61 ± 0.08	0.64 ± 0.10	0.61 ± 0.08	0.36 ± 0.06	0.35 ± 0.05	0.37 ± 0.06	0.35 ± 0.05
Sartorius	0.74 ± 0.19	0.70 ± 0.15	0.66 ± 0.15	0.63 ± 0.13	0.42 ± 0.11	0.40 ± 0.09	0.38 ± 0.08	0.36 ± 0.07
Gracilis	0.64 ± 0.11	0.65 ± 0.10	0.61 ± 0.11	0.60 ± 0.10	0.37 ± 0.07	0.38 ± 0.06	0.35 ± 0.06	0.35 ± 0.06
**Mean ± SD**	**0.66 ± 0.06**	**0.65 ± 0.06**	**0.65 ± 0.05**	**0.63 ± 0.05***	**0.38 ± 0.04**	**0.38 ± 0.03**	**0.38 ± 0.03**	**0.36 ± 0.03***
**Plantar Flexors**								
Gastrocnemius	0.77 ± 0.16	0.80 ± 0.15	0.85 ± 0.20	0.86 ± 0.21	0.44 ± 0.09	0.46 ± 0.09	0.49 ± 0.12	0.49 ± 0.12
Soleus	1.04 ± 0.23	1.09 ± 0.28	1.09 ± 0.22	1.24 ± 0.30	0.60 ± 0.13	0.63 ± 0.16	0.63 ± 0.13	0.72 ± 0.17
Peroneus Longus	0.85 ± 0.19	0.85 ± 0.15	0.83 ± 0.20	0.89 ± 0.20	0.49 ± 0.11	0.49 ± 0.09	0.48 ± 0.11	0.52 ± 0.11
Peroneus Brevis	1.03 ± 0.22	0.97 ± 0.18	0.97 ± 0.19	0.98 ± 0.20	0.59 ± 0.13	0.56 ± 0.10	0.56 ± 0.11	0.56 ± 0.12
Flexor Digitorum	1.02 ± 0.26	1.07 ± 0.28	1.04 ± 0.26	1.23 ± 0.38	0.59 ± 0.15	0.62 ± 0.16	0.60 ± 0.15	0.71 ± 0.22
Flexor Hallucis	1.15 ± 0.22	1.17 ± 0.23	1.20 ± 0.20	1.34 ± 0.28	0.66 ± 0.13	0.67 ± 0.13	0.69 ± 0.11	0.77 ± 0.16
Tibialis Posterior	1.13 ± 0.19	1.12 ± 0.23	1.08 ± 0.19	1.09 ± 0.21	0.65 ± 0.11	0.65 ± 0.13	0.62 ± 0.11	0.63 ± 0.12
**Mean ± SD**	**1.00 ± 0.14**	**1.01 ± 0.14**	**1.01 ± 0.13**	**1.09 ± 0.19±**	**0.58 ± 0.08**	**0.58 ± 0.08**	**0.58 ± 0.08**	**0.63 ± 0.11±**
**Dorsiflexors**								
Tibialis Anterior	1.00 ± 0.17	0.96 ± 0.15	1.03 ± 0.18	0.98 ± 0.16	0.58 ± 0.10	0.55 ± 0.09	0.59 ± 0.10	0.56 ± 0.09
Extensor Digitorum	1.21 ± 0.29	1.23 ± 0.39	1.15 ± 0.22	1.18 ± 0.26	0.70 ± 0.17	0.71 ± 0.23	0.66 ± 0.13	0.68 ± 0.15
Extensor Hallucis	1.07 ± 0.18	1.16 ± 0.23	1.04 ± 0.15	1.08 ± 0.15	0.62 ± 0.10	0.67 ± 0.13	0.60 ± 0.09	0.62 ± 0.09
**Mean ± SD**	**1.10 ± 0.11**	**1.12 ± 0.14**	**1.07 ± 0.07**	**1.08 ± 0.10**	**0.63 ± 0.06**	**0.65 ± 0.08**	**0.62 ± 0.04**	**0.62 ± 0.06**

***Indicates significant decrease after tDCS. ±Indicates significant increase after tDCS. Significance was accepted at $p < 0.05$.

Table S8. Changes in glucose uptake heterogeneity (GUh) for the more- and less-affected muscle groups in Subject 4. Rows in bold represent mean and standard deviation of the respective muscle group. A negative number indicates a decrease in GUh in tDCS compared to SHAM.

Muscle	MA (Right) Leg				LA (Left) Leg			
	**SHAM**	**tDCS**	**ΔGUh**	**% GUh Change**	**SHAM**	**tDCS**	**ΔGUh**	**% GUh Change**
**Knee Extensors**								
Rectus Femoris	23.98	25.42	1.44	6.02	22.04	21.74	-0.30	-1.34
Vastus Medialis	22.56	23.54	0.98	4.35	23.18	23.96	0.77	3.34
Vastus Intermedius	13.87	15.25	1.39	10.02	13.70	14.85	1.14	8.34
Vastus Lateralis	17.61	17.27	-0.34	-1.93	15.90	15.50	-0.40	-2.52
**Mean ± SD**	**19.50 ± 4.65**	**20.37 ± 4.88**	**0.87 ± 0.83**	**4.61 ± 4.97**	**18.71 ± 4.62**	**19.01 ± 4.53**	**0.31 ± 0.77**	**1.96 ± 4.95**
**Knee Flexors**								
BF – Long Head	17.98	18.34	0.36	2.02	17.98	16.79	-1.19	-6.63
BF – Short Head	11.99	13.10	1.11	9.25	14.11	11.48	-2.64	-18.70
Semimembranosus	14.36	12.50	-1.86	-12.98	14.22	13.87	-0.35	-2.46
Semitendinosus	16.10	12.77	-3.33	-20.67	15.34	13.79	-1.54	-10.05
Sartorius	25.82	21.24	-4.58	-17.75	22.02	20.24	-1.79	-8.12
Gracilis	17.81	15.82	-1.99	-11.15	18.15	17.22	-0.93	-5.14
**Mean ± SD**	**17.34 ± 4.72**	**15.63 ± 3.56**	**-1.71 ± 2.15**	**-8.55 ± 13.02**	**16.97 ± 3.04**	**15.56 ± 3.12***	**-1.41 ± 0.78**	**-8.52 ± 6.00**
**Plantar Flexors**								
Gastrocnemius	20.80	19.05	-1.75	-8.41	23.86	24.11	0.26	1.08
Soleus	21.88	25.21	3.33	15.22	19.88	23.84	3.97	19.95
Peroneus Longus	21.87	17.91	-3.96	-18.12	23.51	22.28	-1.24	-5.27
Peroneus Brevis	21.72	18.67	-3.05	-14.04	19.52	20.65	1.14	5.82
Flexor Digitorum	25.37	26.38	1.01	3.97	24.60	31.03	6.43	26.14
Flexor Hallucis	19.14	19.94	0.80	4.19	16.63	20.68	4.05	24.37
Tibialis Posterior	16.78	20.55	3.77	22.48	17.20	18.93	1.74	10.11
**Mean ± SD**	**21.08 ± 2.66**	**21.10 ± 3.33**	**0.02 ± 3.03**	**0.75 ± 15.07**	**20.74 ± 3.27**	**23.08 ± 3.96**	**2.33 ± 2.62**	**11.74 ± 12.07**
**Dorsiflexors**								
Tibialis Anterior	17.17	15.42	-1.74	-10.16	17.21	15.91	-1.30	-7.53
Extensor Digitorum	24.34	31.73	7.39	30.34	19.45	21.53	2.08	10.69
Extensor Hallucis	16.65	19.86	3.21	19.28	14.37	14.02	-0.35	-2.45
**Mean ± SD**	**19.39 ± 4.30**	**22.34 ± 8.43**	**2.95 ± 4.57**	**13.15 ± 20.94**	**17.01 ± 2.55**	**17.15 ± 3.91**	**0.14 ± 1.74**	**0.24 ± 9.41**

*Indicates significant decrease in GUh after tDCS. Significance was accepted at $p < 0.05$.

FOR IRB USE ONLY
APPROVED BY: IRB-01
IRB ID #: 201905825
APPROVAL DATE: 11/18/19

INFORMED CONSENT DOCUMENT

Project Title: **Transcranial Direct Current Stimulation to reduce asymmetric glucose uptake in leg muscles of persons with Multiple Sclerosis**

Principal Investigator: Thorsten Rudroff, PhD

Research Team Contact:

Thorsten Rudroff, PhD (319) 467-0363
Laura Ponto, PhD (319) 356-1857
Craig D. Workman, PhD (319) 467-0746

This consent form describes the research study to help you decide if you want to participate. This form provides important information about what you will be asked to do during the study, about the risks and benefits of the study, and about your rights as a research subject.

- If you have any questions about or do not understand something in this form, you should ask the research team for more information.
- You should discuss your participation with anyone you choose such as family or friends.
- Do not agree to participate in this study unless the research team has answered your questions and you decide that you want to be part of this study.

WHAT IS THE PURPOSE OF THIS STUDY?

This is a research study. We are inviting you to participate in this research study because you have been diagnosed with Multiple Sclerosis (MS).

The purpose of this research study is to investigate the effects of transcranial direct current stimulation (tDCS) on walking ability and the energy usage of the legs of people with MS. We will determine which of your legs is weaker with an isokinetic device. This specialized apparatus provides variable resistance to a movement, so that no matter how much effort you exert, the movement takes place at a constant speed. We will also administer either active tDCS (wearing the device with active stimulation) or sham tDCS (wearing the device without active stimulation) over the part of your brain that controls your weaker leg on two different days separated by at least 5 days.

tDCS is a non-invasive technique in which a very weak electrical current is applied to the head in order to stimulate the brain. To accomplish this, we will place electrodes soaked in salt water over your scalp and forehead. This means that one of the electrodes will be placed over your hair, but no additional substances will be put in your hair and no additional preparation of the hair is necessary. The electrodes will be held in place with a headband. A small current will be passed between the electrodes. You may feel some tingling under the electrodes when the current is first turned on, but no other adverse effects are expected. The stimulation will last for no longer than 10 min.

HOW MANY PEOPLE WILL PARTICIPATE?

Approximately 20 people will take part in this study conducted by investigators at the University of Iowa.

HOW LONG WILL I BE IN THIS STUDY?

If you agree to take part in this study, you will be asked to complete three total study sessions. Each session will be separated by at least 5 days. The first session, which will take place at the Integrative Neurophysiology Laboratory (INPL), will involve muscle strength testing and completing a few questionnaires. This visit is expected to take approximately 1 hour. Sessions 2 and 3, which will take place at the Positron Emission Tomography Imaging Center (PET Imaging Center) and the PET Imaging Lab at Pappajohn Biomedical Discovery Building, will involve walking on a treadmill for 20 minutes in conjunction with either active or sham tDCS. During this time, we will inject you with a radiotracer called [^{18}F]Fluorodeoxyglucose or FDG that will allow us to examine the energy use of your muscles during walking. After you walk on the treadmill, you will undergo PET imaging of your entire body. These sessions will take approximately 1.5 to 2 hours.

WHAT WILL HAPPEN DURING THIS STUDY?

Upon arrival to the INPL for your first session, the protocol procedures will be explained to you. If you agree to participate and sign this consent document, you will complete the Patient Determine Disease Steps and Fatigue Severity Scale questionnaires. You will then perform the leg strength assessment to determine your weaker leg. For Sessions 2 and 3, you will go to the PET Imaging Center and will be taken to the PET Imaging Lab at Pappajohn Biomedical Discovery Building. Next, we will place the two tDCS electrodes on your head; one over the part of your brain that controls your weaker leg and the other above your eyebrow on the other side of your head. Once these electrodes are secured in their place, we will randomly administer active (device on; 3 mA) or sham (device off; 0 mA) tDCS for 10 minutes. We will not tell you if the device is on or off. Once the stimulation is complete, you will remain seated for an additional 10 minutes before you walk on a treadmill for 20 minutes at a speed of your choice. You will walk at the same speed for both sessions and we will ask you to rate how much exertion (effort) you feel it took to complete the walk. Immediately after you have completed the walk, you will be positioned in the PET scanner and a full-body scan will be performed to determine how the muscles of your body have used energy (glucose) during the walk.

PET Scans

This study involves an imaging technique called positron emission tomography (PET). In this scan, the PET scanner takes pictures of the function of your brain and muscles using a radioactive form of sugar called [^{18}F]fluorodeoxyglucose or FDG to determine which parts of the body are most active.

For sessions 2 and 3, the same procedures will be done. You will need to fast for a minimum of 6 hours before the FDG scan.

When you first arrive at the PET Imaging Center, we will measure your height and weight while clothed. On the day that you are going to have the FDG imaging, we will check your blood sugar to make sure that it is at a level of 200 mg/dL or lower. If your blood sugar is higher than 200 mg/dL, we will not be able to do the FDG scan on that day. If you are able to proceed with the scan a small thin tube (catheter) will be placed in a vein that will be used for injecting the PET radiotracer, FDG. We will take you over to the PET lab in the Pappajohn Biomedical Discovery Building where you will be asked to use the restroom and empty your bladder before you will walk on the treadmill and be imaged in the PET scanner.

The PET scanner is a large circular ring of detectors that forms a donut shape. You will be lying on a

bed that can be moved into the middle of this donut during imaging (i.e., taking the pictures). Initially, we will take a CT image to use to correct the subsequent PET images and then we will take the PET images of your entire body (top of your head to your toes). It is important that you lie quietly and not move during imaging.

When the imaging is complete, we will remove the IV catheter and ask you to go to the restroom again before you leave the PET Imaging Center. Each PET scan will last approximately 2 hours.

Tissue/Blood/Data Storage for Future Use

As part of this study, we are obtaining PET images from you. We will study your PET images in the future, after this study is over. Your sample, information, and/or data may be placed in a central repository or other national repositories sponsored by the National Institutes of Health or other Federal agencies. If this happens, it may be stripped of identifiers (such as name, date of birth, address, etc). Other qualified researchers who obtain proper permission may gain access to your sample and/or data for use in approved research studies that may or may not be related to in the purpose of this study.

The tests we might want to use to study your PET images may not even exist at this time. Therefore, we are asking for your permission to store your PET images so that we can study them in the future. These future studies may provide additional information that will be helpful in understanding Multiple Sclerosis, but it is unlikely that what we learn from these studies will have a direct benefit to you. It is possible that your PET images might be used to develop products tests, or discoveries that could be patented and licensed. In some instances, these may have potential commercial value and may be developed by the Investigators, University of Iowa, commercial companies, organizations funding this research, or others that may not be working directly with this research team. However, donors of PET images do not retain any property rights to the materials. Therefore, there are no plans to provide financial compensation to you should this occur.

Your PET images will be stored *with a code which may be linked to* your age (date of birth), height, weight, Multiple Sclerosis diagnosis, and the date the images were obtained. If you agree now to future use of your PET images but decide in the future that you would like to have it removed from future research, you should contact Dr. Thorsten Rudroff by phone at 319-467-0363. However, if some research with your PET images has already been completed, the information from that research may still be used.

WILL I BE NOTIFIED IF MY IMAGES RESULT IN AN UNEXPECTED FINDING?

The results from the images we collect in this research study are not the same quality as what you would receive as part of your routine health care. The image results will not be reviewed by a physician who normally reads such results. Due to this, you will not be informed of any unexpected findings. The results of your images will not be placed in your medical record with your primary care physician or otherwise. If you believe you are having symptoms that may require care, you should contact your primary care physician.

WHAT ARE THE RISKS OF THIS STUDY?

You may experience one or more of the risks indicated below from being in this study. In addition to

these, there may be other unknown risks, or risks that we did not anticipate, associated with being in this study.

Risks of tDCS

tDCS has been conducted on humans and animals for many years and no evidence has emerged to suggest that it is harmful or has ever induced a serious side effect. However, the safety of tDCS is dependent upon current strength, electrode size, and stimulation duration. We will follow the existing guidelines for safe and effective stimulation that have been identified for research studies and in clinical practice for tDCS applications. In the several hundred studies conducted on humans using tDCS, the only side effects that have been reported when proper guidelines are followed are temporary tingling, itching, mild headache, or skin redness under the electrodes in some subjects.

Risks of PET Imaging

This study involves the administration of a PET radiopharmaceutical, [^{18}F]fluorodeoxyglucose or FDG. FDG has been used at the University of Iowa and throughout the world with no known adverse reactions reported in the medical literature. The only discomfort during the PET scans will be from lying still in the confined space of the scanner for the required length of time, fasting for 6 hours, and that associated with the venous catheter necessary for this imaging study.

Fasting

We will ask you to fast for a minimum of 6 hours before the FDG study. We will check your blood sugar using blood from a finger stick before we inject the FDG. If your blood sugar is too low or if you feel like your blood sugar is too low at any time during the study, we can provide you with juice.

Radiation Risks

The ^{18}F-FDG is a radiotracer and therefore, you will be receiving a dose of radiation from your participation in this research. The maximum amount of radiation from the research-related radiation procedures in this study is equivalent to approximately 92% of the annual radiation limit for a medical worker. Long-term effects on your health, such as cancer, cannot be ruled out from this amount of radiation. This dose estimate takes into account only the exposure to research procedures in this project. If you have participated in other research studies involving radiation exposure, you should be aware that the risk of effects from radiation is believed to increase with each exposure you receive (including studies performed as part of your medical care).

Radiation Exposure in Women Capable of Becoming Pregnant

You may not participate in this study if you are pregnant. If you are capable of becoming pregnant, we will perform a pregnancy test before exposure to research-related radiation. You must tell us if you may have become pregnant within the previous 14 days because the pregnancy test is unreliable during that time.

Insertion of catheter (tube) into an arm vein (during PET scan)

A catheter (tube) will be inserted into a vein to allow us to give you the FDG. As in any procedure involving catheters placed in veins, there are several risks, including infection, clotting or continued bleeding. There may also be bruising, skin irritation and a dull ache at the site of insertion or a feeling of dizziness or fainting upon placement. These risks will be minimized by having only medical personnel trained in the placement of venous catheters inserting the line.

FOR IRB USE ONLY
APPROVED BY: IRB-01
IRB ID #: 201905825
APPROVAL DATE: 11/18/19

WHAT ARE THE BENEFITS OF THIS STUDY?

You will not benefit from being in this study. However, we hope that, in the future, other people might benefit from this study because this study is designed for the researchers to learn more about how tDCS may improve the treatment of the debilitating symptoms of a variety of diseases, including Multiple Sclerosis.

WILL IT COST ME ANYTHING TO BE IN THIS STUDY?

You will be responsible for any parking and/or transportation costs associated with your visits. You will not have any other costs for being in this research study. All procedures and tests will be provided at no cost to you. You and/or your medical/hospital insurance carrier will remain responsible for your regular medical care expenses.

WILL I BE PAID FOR PARTICIPATING?

You will be paid for being in this research study. You will receive $20 for the first visit, $40 for the second visit, and $40 for the third visit ($100 total).

WHO IS FUNDING THIS STUDY?

The University and the research team are receiving no payments from other agencies, organizations, or companies to conduct this research study.

WHAT IF I AM INJURED AS A RESULT OF THIS STUDY?

- If you are injured or become ill from taking part in this study, medical treatment is available at the University of Iowa Hospitals and Clinics.
- The University of Iowa does not plan to provide free medical care or payment for treatment of any illness or injury resulting from this study unless it is the direct result of proven negligence by a University employee.
- If you experience a research-related illness or injury, you and/or your medical or hospital insurance carrier will be responsible for the cost of treatment.

WILL YOU KEEP MY NAME ON FILE TO GIVE TO OTHERS?

We will keep information about you in a special kind of computer listing called a registry. A registry keeps information about you on file so that *other* researchers, not involved in this particular study, may contact you in the future about whether you are interested in being in *different* research studies. The registry will contain information such as your name, address, age, and selected other information such as gender, weight and height. We will keep the information in this registry secure by on a password-protected computer in a restricted-access office. You may request that your personal information be removed from this file at any time by contacting Thorsten Rudroff at (319) 467-0746 or by writing to him at E432 FH, University of Iowa, Iowa City Iowa 52242. If you choose to do this, your information will be permanently removed from our contact list.

WHAT ABOUT CONFIDENTIALITY?

We will keep your participation in this research study confidential to the extent permitted by law. However, it is possible that other people such as those indicated below may become aware of your participation in this study and may inspect and copy records pertaining to this research. Some of these records could contain information that personally identifies you.

- Federal government regulatory agencies,
- The U.S. Food and Drug Administration
- Auditing departments of the University of Iowa, and
- The University of Iowa Institutional Review Board (a committee that reviews and approves research studies)

To help protect your confidentiality, we will keep private all research records that identify you. Your data will be coded to remove any direct links to your identity, and any identifying material will be locked in a secure filing cabinet. If we write a report or article about this study or share the study data set with others, we will do so in such a way that you cannot be directly identified.

The University of Iowa Hospitals and Clinics generally requires that we document your participation in research occurring in a University of Iowa Health Care facility. This documentation will be in either your medical record or a database maintained on behalf of the institution reflecting that you are participating in this study. The information included will provide contact information for the research team as well as information about the risks associated with this study. We will keep this Informed Consent Document in our research files; it will not be placed in your medical record chart.

WILL MY HEALTH INFORMATION BE USED DURING THIS STUDY?

The Federal Health Insurance Portability and Accountability Act (HIPAA) requires University of Iowa Health Care to obtain your permission for the research team to access or create "protected health information" about you for purposes of this research study. Protected health information is information that personally identifies you and relates to your past, present, or future physical or mental health condition or care. We will access or create health information about you, as described in this document, for purposes of this research study. Once your health care provider has disclosed your protected health information to us, it may no longer be protected by the Federal HIPAA privacy regulations, but we will continue to protect your confidentiality as described under "Confidentiality."

We may share your health information related to this study with other parties including federal government regulatory agencies, the University of Iowa Institutional Review Boards and support staff.

You cannot participate in this study unless you permit us to use your protected health information. If you choose *not* to allow us to use your protected health information, we will discuss any non-research alternatives available to you. Your decision will not affect your right to medical care that is not research-related. Your signature on this Consent Document authorizes your health care provider to give us permission to use or create health information about you.

Although you may not be allowed to see study information until after this study is over, you may be given access to your health care records by contacting your health care provider. Your permission for us

to access or create protected health information about you for purposes of this study has no expiration date. You may withdraw your permission for us to use your health information for this research study by sending a written notice to Thorsten Rudroff, Department of Health & Human Physiology, University of Iowa, E432, Field House, Iowa City, Iowa 52242. However, we may still use your health information that was collected before withdrawing your permission. Also, if we have sent your health information to a third party, such as the study sponsor, or we have removed your identifying information, it may not be possible to prevent its future use. You will receive a copy of this signed document.

IS BEING IN THIS STUDY VOLUNTARY?

Taking part in this research study is completely voluntary. You may choose not to take part at all. If you decide to be in this study, you may stop participating at any time. If you decide not to be in this study, or if you stop participating at any time, you won't be penalized or lose any benefits for which you otherwise qualify.

What if I Decide to Drop Out of the Study?
Your participation in this research is voluntary. If you decide to participate, you may withdraw your consent and stop participating at any time.

Will I Receive New Information About the Study while Participating?
If we obtain any new information during this study that might affect your willingness to continue participating in the study, we'll promptly provide you with that information.

Can Someone Else End my Participation in this Study?
Under certain circumstances, the researchers might decide to end your participation in this research study earlier than planned. This might happen because in our judgment it would not be safe or comfortable for you to continue.

WHAT IF I HAVE QUESTIONS?

We encourage you to ask questions. If you have any questions about the research study itself, please contact: Thorsten Rudroff, (319) 467-0363. If you experience a research-related injury, please contact: Thorsten Rudroff, (319) 467-0363.

If you have questions, concerns, or complaints about your rights as a research subject or about research related injury, please contact the Human Subjects Office, 105 Hardin Library for the Health Sciences, 600 Newton Rd, The University of Iowa, Iowa City, IA 52242-1098, (319) 335-6564, or e-mail irb@uiowa.edu. General information about being a research subject can be found by clicking "Info for Public" on the Human Subjects Office web site, http://hso.research.uiowa.edu/. To offer input about your experiences as a research subject or to speak to someone other than the research staff, call the Human Subjects Office at the number above.

This Informed Consent Document is not a contract. It is a written explanation of what will happen during the study if you decide to participate. You are not waiving any legal rights by signing this Informed Consent Document. Your signature indicates that this research study has been explained to you, that your questions have been answered, and that you agree to take part in this study. You will receive a copy of this form.

FOR IRB USE ONLY
APPROVED BY: IRB-01
IRB ID #: 201905825
APPROVAL DATE: 11/18/19

University of Iowa
CONFIDENTIAL

Subject's Name (printed): S[illegible]ombs

University of Iowa
CONFIDENTIAL

[illegible] ______ 7-23-2020
(Signature of Subject) (Date)

Statement of Person Who Obtained Consent

I have discussed the above points with the subject or, where appropriate, with the subject's legally authorized representative. It is my opinion that the subject understands the risks, benefits, and procedures involved with participation in this research study.

Alexandra Fiertram ______ 7/23/20
(Signature of Person who Obtained Consent) (Date)

FOR IRB USE ONLY
APPROVED BY: IRB-01
IRB ID #: 201905825
APPROVAL DATE: 11/18/19

INFORMED CONSENT DOCUMENT

Project Title: **Transcranial Direct Current Stimulation to reduce asymmetric glucose uptake in leg muscles of persons with Multiple Sclerosis**

Principal Investigator: Thorsten Rudroff, PhD

Research Team Contact:

Thorsten Rudroff, PhD	(319) 467-0363
Laura Ponto, PhD	(319) 356-1857
Craig D. Workman, PhD	(319) 467-0746

This consent form describes the research study to help you decide if you want to participate. This form provides important information about what you will be asked to do during the study, about the risks and benefits of the study, and about your rights as a research subject.

- If you have any questions about or do not understand something in this form, you should ask the research team for more information.
- You should discuss your participation with anyone you choose such as family or friends.
- Do not agree to participate in this study unless the research team has answered your questions and you decide that you want to be part of this study.

WHAT IS THE PURPOSE OF THIS STUDY?

This is a research study. We are inviting you to participate in this research study because you have been diagnosed with Multiple Sclerosis (MS).

The purpose of this research study is to investigate the effects of transcranial direct current stimulation (tDCS) on walking ability and the energy usage of the legs of people with MS. We will determine which of your legs is weaker with an isokinetic device. This specialized apparatus provides variable resistance to a movement, so that no matter how much effort you exert, the movement takes place at a constant speed. We will also administer either active tDCS (wearing the device with active stimulation) or sham tDCS (wearing the device without active stimulation) over the part of your brain that controls your weaker leg on two different days separated by at least 5 days.

tDCS is a non-invasive technique in which a very weak electrical current is applied to the head in order to stimulate the brain. To accomplish this, we will place electrodes soaked in salt water over your scalp and forehead. This means that one of the electrodes will be placed over your hair, but no additional substances will be put in your hair and no additional preparation of the hair is necessary. The electrodes will be held in place with a headband. A small current will be passed between the electrodes. You may feel some tingling under the electrodes when the current is first turned on, but no other adverse effects are expected. The stimulation will last for no longer than 10 min.

HOW MANY PEOPLE WILL PARTICIPATE?

Approximately 20 people will take part in this study conducted by investigators at the University of Iowa.

HOW LONG WILL I BE IN THIS STUDY?

If you agree to take part in this study, you will be asked to complete three total study sessions. Each session will be separated by at least 5 days. The first session, which will take place at the Integrative Neurophysiology Laboratory (INPL), will involve muscle strength testing and completing a few questionnaires. This visit is expected to take approximately 1 hour. Sessions 2 and 3, which will take place at the Positron Emission Tomography Imaging Center (PET Imaging Center) and the PET Imaging Lab at Pappajohn Biomedical Discovery Building, will involve walking on a treadmill for 20 minutes in conjunction with either active or sham tDCS. During this time, we will inject you with a radiotracer called [^{18}F]Fluorodeoxyglucose or FDG that will allow us to examine the energy use of your muscles during walking. After you walk on the treadmill, you will undergo PET imaging of your entire body. These sessions will take approximately 1.5 to 2 hours.

WHAT WILL HAPPEN DURING THIS STUDY?

Upon arrival to the INPL for your first session, the protocol procedures will be explained to you. If you agree to participate and sign this consent document, you will complete the Patient Determine Disease Steps and Fatigue Severity Scale questionnaires. You will then perform the leg strength assessment to determine your weaker leg. For Sessions 2 and 3, you will go to the PET Imaging Center and will be taken to the PET Imaging Lab at Pappajohn Biomedical Discovery Building. Next, we will place the two tDCS electrodes on your head; one over the part of your brain that controls your weaker leg and the other above your eyebrow on the other side of your head. Once these electrodes are secured in their place, we will randomly administer active (device on; 3 mA) or sham (device off; 0 mA) tDCS for 10 minutes. We will not tell you if the device is on or off. Once the stimulation is complete, you will remain seated for an additional 10 minutes before you walk on a treadmill for 20 minutes at a speed of your choice. You will walk at the same speed for both sessions and we will ask you to rate how much exertion (effort) you feel it took to complete the walk. Immediately after you have completed the walk, you will be positioned in the PET scanner and a full-body scan will be performed to determine how the muscles of your body have used energy (glucose) during the walk.

PET Scans

This study involves an imaging technique called positron emission tomography (PET). In this scan, the PET scanner takes pictures of the function of your brain and muscles using a radioactive form of sugar called [^{18}F]fluorodeoxyglucose or FDG to determine which parts of the body are most active.

For sessions 2 and 3, the same procedures will be done. You will need to fast for a minimum of 6 hours before the FDG scan.

When you first arrive at the PET Imaging Center, we will measure your height and weight while clothed. On the day that you are going to have the FDG imaging, we will check your blood sugar to make sure that it is at a level of 200 mg/dL or lower. If your blood sugar is higher than 200 mg/dL, we will not be able to do the FDG scan on that day. If you are able to proceed with the scan a small thin tube (catheter) will be placed in a vein that will be used for injecting the PET radiotracer, FDG. We will take you over to the PET lab in the Pappajohn Biomedical Discovery Building where you will be asked to use the restroom and empty your bladder before you will walk on the treadmill and be imaged in the PET scanner.

The PET scanner is a large circular ring of detectors that forms a donut shape. You will be lying on a

bed that can be moved into the middle of this donut during imaging (i.e., taking the pictures). Initially, we will take a CT image to use to correct the subsequent PET images and then we will take the PET images of your entire body (top of your head to your toes). It is important that you lie quietly and not move during imaging.

When the imaging is complete, we will remove the IV catheter and ask you to go to the restroom again before you leave the PET Imaging Center. Each PET scan will last approximately 2 hours.

Tissue/Blood/Data Storage for Future Use

As part of this study, we are obtaining PET images from you. We will study your PET images in the future, after this study is over. Your sample, information, and/or data may be placed in a central repository or other national repositories sponsored by the National Institutes of Health or other Federal agencies. If this happens, it may be stripped of identifiers (such as name, date of birth, address, etc). Other qualified researchers who obtain proper permission may gain access to your sample and/or data for use in approved research studies that may or may not be related to in the purpose of this study.

The tests we might want to use to study your PET images may not even exist at this time. Therefore, we are asking for your permission to store your PET images so that we can study them in the future. These future studies may provide additional information that will be helpful in understanding Multiple Sclerosis, but it is unlikely that what we learn from these studies will have a direct benefit to you. It is possible that your PET images might be used to develop products tests, or discoveries that could be patented and licensed. In some instances, these may have potential commercial value and may be developed by the Investigators, University of Iowa, commercial companies, organizations funding this research, or others that may not be working directly with this research team. However, donors of PET images do not retain any property rights to the materials. Therefore, there are no plans to provide financial compensation to you should this occur.

Your PET images will be stored *with a code which may be linked to* your age (date of birth), height, weight, Multiple Sclerosis diagnosis, and the date the images were obtained. If you agree now to future use of your PET images but decide in the future that you would like to have it removed from future research, you should contact Dr. Thorsten Rudroff by phone at 319-467-0363. However, if some research with your PET images has already been completed, the information from that research may still be used.

WILL I BE NOTIFIED IF MY IMAGES RESULT IN AN UNEXPECTED FINDING?

The results from the images we collect in this research study are not the same quality as what you would receive as part of your routine health care. The image results will not be reviewed by a physician who normally reads such results. Due to this, you will not be informed of any unexpected findings. The results of your images will not be placed in your medical record with your primary care physician or otherwise. If you believe you are having symptoms that may require care, you should contact your primary care physician.

WHAT ARE THE RISKS OF THIS STUDY?

You may experience one or more of the risks indicated below from being in this study. In addition to

these, there may be other unknown risks, or risks that we did not anticipate, associated with being in this study.

Risks of tDCS

tDCS has been conducted on humans and animals for many years and no evidence has emerged to suggest that it is harmful or has ever induced a serious side effect. However, the safety of tDCS is dependent upon current strength, electrode size, and stimulation duration. We will follow the existing guidelines for safe and effective stimulation that have been identified for research studies and in clinical practice for tDCS applications. In the several hundred studies conducted on humans using tDCS, the only side effects that have been reported when proper guidelines are followed are temporary tingling, itching, mild headache, or skin redness under the electrodes in some subjects.

Risks of PET Imaging

This study involves the administration of a PET radiopharmaceutical, [^{18}F]fluorodeoxyglucose or FDG. FDG has been used at the University of Iowa and throughout the world with no known adverse reactions reported in the medical literature. The only discomfort during the PET scans will be from lying still in the confined space of the scanner for the required length of time, fasting for 6 hours, and that associated with the venous catheter necessary for this imaging study.

Fasting

We will ask you to fast for a minimum of 6 hours before the FDG study. We will check your blood sugar using blood from a finger stick before we inject the FDG. If your blood sugar is too low or if you feel like your blood sugar is too low at any time during the study, we can provide you with juice.

Radiation Risks

The ^{18}F-FDG is a radiotracer and therefore, you will be receiving a dose of radiation from your participation in this research. The maximum amount of radiation from the research-related radiation procedures in this study is equivalent to approximately 92% of the annual radiation limit for a medical worker. Long-term effects on your health, such as cancer, cannot be ruled out from this amount of radiation. This dose estimate takes into account only the exposure to research procedures in this project. If you have participated in other research studies involving radiation exposure, you should be aware that the risk of effects from radiation is believed to increase with each exposure you receive (including studies performed as part of your medical care).

Radiation Exposure in Women Capable of Becoming Pregnant

You may not participate in this study if you are pregnant. If you are capable of becoming pregnant, we will perform a pregnancy test before exposure to research-related radiation. You must tell us if you may have become pregnant within the previous 14 days because the pregnancy test is unreliable during that time.

Insertion of catheter (tube) into an arm vein (during PET scan)

A catheter (tube) will be inserted into a vein to allow us to give you the FDG. As in any procedure involving catheters placed in veins, there are several risks, including infection, clotting or continued bleeding. There may also be bruising, skin irritation and a dull ache at the site of insertion or a feeling of dizziness or fainting upon placement. These risks will be minimized by having only medical personnel trained in the placement of venous catheters inserting the line.

WHAT ARE THE BENEFITS OF THIS STUDY?

You will not benefit from being in this study. However, we hope that, in the future, other people might benefit from this study because this study is designed for the researchers to learn more about how tDCS may improve the treatment of the debilitating symptoms of a variety of diseases, including Multiple Sclerosis.

WILL IT COST ME ANYTHING TO BE IN THIS STUDY?

You will be responsible for any parking and/or transportation costs associated with your visits. You will not have any other costs for being in this research study. All procedures and tests will be provided at no cost to you. You and/or your medical/hospital insurance carrier will remain responsible for your regular medical care expenses.

WILL I BE PAID FOR PARTICIPATING?

You will be paid for being in this research study. You will receive $20 for the first visit, $40 for the second visit, and $40 for the third visit ($100 total).

WHO IS FUNDING THIS STUDY?

The University and the research team are receiving no payments from other agencies, organizations, or companies to conduct this research study.

WHAT IF I AM INJURED AS A RESULT OF THIS STUDY?

- If you are injured or become ill from taking part in this study, medical treatment is available at the University of Iowa Hospitals and Clinics.
- The University of Iowa does not plan to provide free medical care or payment for treatment of any illness or injury resulting from this study unless it is the direct result of proven negligence by a University employee.
- If you experience a research-related illness or injury, you and/or your medical or hospital insurance carrier will be responsible for the cost of treatment.

WILL YOU KEEP MY NAME ON FILE TO GIVE TO OTHERS?

We will keep information about you in a special kind of computer listing called a registry. A registry keeps information about you on file so that *other* researchers, not involved in this particular study, may contact you in the future about whether you are interested in being in *different* research studies. The registry will contain information such as your name, address, age, and selected other information such as gender, weight and height. We will keep the information in this registry secure by on a password-protected computer in a restricted-access office. You may request that your personal information be removed from this file at any time by contacting Thorsten Rudroff at (319) 467-0746 or by writing to him at E432 FH, University of Iowa, Iowa City Iowa 52242. If you choose to do this, your information will be permanently removed from our contact list.

WHAT ABOUT CONFIDENTIALITY?

We will keep your participation in this research study confidential to the extent permitted by law. However, it is possible that other people such as those indicated below may become aware of your participation in this study and may inspect and copy records pertaining to this research. Some of these records could contain information that personally identifies you.

- Federal government regulatory agencies,
- The U.S. Food and Drug Administration
- Auditing departments of the University of Iowa, and
- The University of Iowa Institutional Review Board (a committee that reviews and approves research studies)

To help protect your confidentiality, we will keep private all research records that identify you. Your data will be coded to remove any direct links to your identity, and any identifying material will be locked in a secure filing cabinet. If we write a report or article about this study or share the study data set with others, we will do so in such a way that you cannot be directly identified.

The University of Iowa Hospitals and Clinics generally requires that we document your participation in research occurring in a University of Iowa Health Care facility. This documentation will be in either your medical record or a database maintained on behalf of the institution reflecting that you are participating in this study. The information included will provide contact information for the research team as well as information about the risks associated with this study. We will keep this Informed Consent Document in our research files; it will not be placed in your medical record chart.

WILL MY HEALTH INFORMATION BE USED DURING THIS STUDY?

The Federal Health Insurance Portability and Accountability Act (HIPAA) requires University of Iowa Health Care to obtain your permission for the research team to access or create "protected health information" about you for purposes of this research study. Protected health information is information that personally identifies you and relates to your past, present, or future physical or mental health condition or care. We will access or create health information about you, as described in this document, for purposes of this research study. Once your health care provider has disclosed your protected health information to us, it may no longer be protected by the Federal HIPAA privacy regulations, but we will continue to protect your confidentiality as described under "Confidentiality."

We may share your health information related to this study with other parties including federal government regulatory agencies, the University of Iowa Institutional Review Boards and support staff.

You cannot participate in this study unless you permit us to use your protected health information. If you choose *not* to allow us to use your protected health information, we will discuss any non-research alternatives available to you. Your decision will not affect your right to medical care that is not research-related. Your signature on this Consent Document authorizes your health care provider to give us permission to use or create health information about you.

Although you may not be allowed to see study information until after this study is over, you may be given access to your health care records by contacting your health care provider. Your permission for us

to access or create protected health information about you for purposes of this study has no expiration date. You may withdraw your permission for us to use your health information for this research study by sending a written notice to Thorsten Rudroff, Department of Health & Human Physiology, University of Iowa, E432, Field House, Iowa City, Iowa 52242. However, we may still use your health information that was collected before withdrawing your permission. Also, if we have sent your health information to a third party, such as the study sponsor, or we have removed your identifying information, it may not be possible to prevent its future use. You will receive a copy of this signed document.

IS BEING IN THIS STUDY VOLUNTARY?

Taking part in this research study is completely voluntary. You may choose not to take part at all. If you decide to be in this study, you may stop participating at any time. If you decide not to be in this study, or if you stop participating at any time, you won't be penalized or lose any benefits for which you otherwise qualify.

What if I Decide to Drop Out of the Study?

Your participation in this research is voluntary. If you decide to participate, you may withdraw your consent and stop participating at any time.

Will I Receive New Information About the Study while Participating?

If we obtain any new information during this study that might affect your willingness to continue participating in the study, we'll promptly provide you with that information.

Can Someone Else End my Participation in this Study?

Under certain circumstances, the researchers might decide to end your participation in this research study earlier than planned. This might happen because in our judgment it would not be safe or comfortable for you to continue.

WHAT IF I HAVE QUESTIONS?

We encourage you to ask questions. If you have any questions about the research study itself, please contact: Thorsten Rudroff, (319) 467-0363. If you experience a research-related injury, please contact: Thorsten Rudroff, (319) 467-0363.

If you have questions, concerns, or complaints about your rights as a research subject or about research related injury, please contact the Human Subjects Office, 105 Hardin Library for the Health Sciences, 600 Newton Rd, The University of Iowa, Iowa City, IA 52242-1098, (319) 335-6564, or e-mail irb@uiowa.edu. General information about being a research subject can be found by clicking "Info for Public" on the Human Subjects Office web site, http://hso.research.uiowa.edu/. To offer input about your experiences as a research subject or to speak to someone other than the research staff, call the Human Subjects Office at the number above.

This Informed Consent Document is not a contract. It is a written explanation of what will happen during the study if you decide to participate. You are not waiving any legal rights by signing this Informed Consent Document. Your signature indicates that this research study has been explained to you, that your questions have been answered, and that you agree to take part in this study. You will receive a copy of this form.

FOR IRB USE ONLY
APPROVED BY: IRB-01
IRB ID #: 201905825
APPROVAL DATE: 11/18/19

University of Iowa CONFIDENTIAL

Subject's Name (printed): [illegible]

University of Iowa CONFIDENTIAL

(Sig[illegible]   7/10/20 (Date)

Statement of Person Who Obtained Consent

I have discussed the above points with the subject or, where appropriate, with the subject's legally authorized representative. It is my opinion that the subject understands the risks, benefits, and procedures involved with participation in this research study.

Alexandra Fieth   7/10/20

(Signature of Person who Obtained Consent)   (Date)

FOR IRB USE ONLY
APPROVED BY: IRB-01
IRB ID #: 201905825
APPROVAL DATE: 11/18/19

INFORMED CONSENT DOCUMENT

Project Title: **Transcranial Direct Current Stimulation to reduce asymmetric glucose uptake in leg muscles of persons with Multiple Sclerosis**

Principal Investigator: Thorsten Rudroff, PhD

Research Team Contact:

Thorsten Rudroff, PhD	(319) 467-0363
Laura Ponto, PhD	(319) 356-1857
Craig D. Workman, PhD	(319) 467-0746

This consent form describes the research study to help you decide if you want to participate. This form provides important information about what you will be asked to do during the study, about the risks and benefits of the study, and about your rights as a research subject.

- If you have any questions about or do not understand something in this form, you should ask the research team for more information.
- You should discuss your participation with anyone you choose such as family or friends.
- Do not agree to participate in this study unless the research team has answered your questions and you decide that you want to be part of this study.

WHAT IS THE PURPOSE OF THIS STUDY?

This is a research study. We are inviting you to participate in this research study because you have been diagnosed with Multiple Sclerosis (MS).

The purpose of this research study is to investigate the effects of transcranial direct current stimulation (tDCS) on walking ability and the energy usage of the legs of people with MS. We will determine which of your legs is weaker with an isokinetic device. This specialized apparatus provides variable resistance to a movement, so that no matter how much effort you exert, the movement takes place at a constant speed. We will also administer either active tDCS (wearing the device with active stimulation) or sham tDCS (wearing the device without active stimulation) over the part of your brain that controls your weaker leg on two different days separated by at least 5 days.

tDCS is a non-invasive technique in which a very weak electrical current is applied to the head in order to stimulate the brain. To accomplish this, we will place electrodes soaked in salt water over your scalp and forehead. This means that one of the electrodes will be placed over your hair, but no additional substances will be put in your hair and no additional preparation of the hair is necessary. The electrodes will be held in place with a headband. A small current will be passed between the electrodes. You may feel some tingling under the electrodes when the current is first turned on, but no other adverse effects are expected. The stimulation will last for no longer than 10 min.

HOW MANY PEOPLE WILL PARTICIPATE?

Approximately 20 people will take part in this study conducted by investigators at the University of Iowa.

HOW LONG WILL I BE IN THIS STUDY?

If you agree to take part in this study, you will be asked to complete three total study sessions. Each session will be separated by at least 5 days. The first session, which will take place at the Integrative Neurophysiology Laboratory (INPL), will involve muscle strength testing and completing a few questionnaires. This visit is expected to take approximately 1 hour. Sessions 2 and 3, which will take place at the Positron Emission Tomography Imaging Center (PET Imaging Center) and the PET Imaging Lab at Pappajohn Biomedical Discovery Building, will involve walking on a treadmill for 20 minutes in conjunction with either active or sham tDCS. During this time, we will inject you with a radiotracer called [^{18}F]Fluorodeoxyglucose or FDG that will allow us to examine the energy use of your muscles during walking. After you walk on the treadmill, you will undergo PET imaging of your entire body. These sessions will take approximately 1.5 to 2 hours.

WHAT WILL HAPPEN DURING THIS STUDY?

Upon arrival to the INPL for your first session, the protocol procedures will be explained to you. If you agree to participate and sign this consent document, you will complete the Patient Determine Disease Steps and Fatigue Severity Scale questionnaires. You will then perform the leg strength assessment to determine your weaker leg. For Sessions 2 and 3, you will go to the PET Imaging Center and will be taken to the PET Imaging Lab at Pappajohn Biomedical Discovery Building. Next, we will place the two tDCS electrodes on your head; one over the part of your brain that controls your weaker leg and the other above your eyebrow on the other side of your head. Once these electrodes are secured in their place, we will randomly administer active (device on; 3 mA) or sham (device off; 0 mA) tDCS for 10 minutes. We will not tell you if the device is on or off. Once the stimulation is complete, you will remain seated for an additional 10 minutes before you walk on a treadmill for 20 minutes at a speed of your choice. You will walk at the same speed for both sessions and we will ask you to rate how much exertion (effort) you feel it took to complete the walk. Immediately after you have completed the walk, you will be positioned in the PET scanner and a full-body scan will be performed to determine how the muscles of your body have used energy (glucose) during the walk.

PET Scans

This study involves an imaging technique called positron emission tomography (PET). In this scan, the PET scanner takes pictures of the function of your brain and muscles using a radioactive form of sugar called [^{18}F]fluorodeoxyglucose or FDG to determine which parts of the body are most active.

For sessions 2 and 3, the same procedures will be done. You will need to fast for a minimum of 6 hours before the FDG scan.

When you first arrive at the PET Imaging Center, we will measure your height and weight while clothed. On the day that you are going to have the FDG imaging, we will check your blood sugar to make sure that it is at a level of 200 mg/dL or lower. If your blood sugar is higher than 200 mg/dL, we will not be able to do the FDG scan on that day. If you are able to proceed with the scan a small thin tube (catheter) will be placed in a vein that will be used for injecting the PET radiotracer, FDG. We will take you over to the PET lab in the Pappajohn Biomedical Discovery Building where you will be asked to use the restroom and empty your bladder before you will walk on the treadmill and be imaged in the PET scanner.

The PET scanner is a large circular ring of detectors that forms a donut shape. You will be lying on a

bed that can be moved into the middle of this donut during imaging (i.e., taking the pictures). Initially, we will take a CT image to use to correct the subsequent PET images and then we will take the PET images of your entire body (top of your head to your toes). It is important that you lie quietly and not move during imaging.

When the imaging is complete, we will remove the IV catheter and ask you to go to the restroom again before you leave the PET Imaging Center. Each PET scan will last approximately 2 hours.

Tissue/Blood/Data Storage for Future Use

As part of this study, we are obtaining PET images from you. We will study your PET images in the future, after this study is over. Your sample, information, and/or data may be placed in a central repository or other national repositories sponsored by the National Institutes of Health or other Federal agencies. If this happens, it may be stripped of identifiers (such as name, date of birth, address, etc). Other qualified researchers who obtain proper permission may gain access to your sample and/or data for use in approved research studies that may or may not be related to in the purpose of this study.

The tests we might want to use to study your PET images may not even exist at this time. Therefore, we are asking for your permission to store your PET images so that we can study them in the future. These future studies may provide additional information that will be helpful in understanding Multiple Sclerosis, but it is unlikely that what we learn from these studies will have a direct benefit to you. It is possible that your PET images might be used to develop products tests, or discoveries that could be patented and licensed. In some instances, these may have potential commercial value and may be developed by the Investigators, University of Iowa, commercial companies, organizations funding this research, or others that may not be working directly with this research team. However, donors of PET images do not retain any property rights to the materials. Therefore, there are no plans to provide financial compensation to you should this occur.

Your PET images will be stored *with a code which may be linked to* your age (date of birth), height, weight, Multiple Sclerosis diagnosis, and the date the images were obtained. If you agree now to future use of your PET images but decide in the future that you would like to have it removed from future research, you should contact Dr. Thorsten Rudroff by phone at 319-467-0363. However, if some research with your PET images has already been completed, the information from that research may still be used.

WILL I BE NOTIFIED IF MY IMAGES RESULT IN AN UNEXPECTED FINDING?

The results from the images we collect in this research study are not the same quality as what you would receive as part of your routine health care. The image results will not be reviewed by a physician who normally reads such results. Due to this, you will not be informed of any unexpected findings. The results of your images will not be placed in your medical record with your primary care physician or otherwise. If you believe you are having symptoms that may require care, you should contact your primary care physician.

WHAT ARE THE RISKS OF THIS STUDY?

You may experience one or more of the risks indicated below from being in this study. In addition to

these, there may be other unknown risks, or risks that we did not anticipate, associated with being in this study.

Risks of tDCS

tDCS has been conducted on humans and animals for many years and no evidence has emerged to suggest that it is harmful or has ever induced a serious side effect. However, the safety of tDCS is dependent upon current strength, electrode size, and stimulation duration. We will follow the existing guidelines for safe and effective stimulation that have been identified for research studies and in clinical practice for tDCS applications. In the several hundred studies conducted on humans using tDCS, the only side effects that have been reported when proper guidelines are followed are temporary tingling, itching, mild headache, or skin redness under the electrodes in some subjects.

Risks of PET Imaging

This study involves the administration of a PET radiopharmaceutical, [^{18}F]fluorodeoxyglucose or FDG. FDG has been used at the University of Iowa and throughout the world with no known adverse reactions reported in the medical literature. The only discomfort during the PET scans will be from lying still in the confined space of the scanner for the required length of time, fasting for 6 hours, and that associated with the venous catheter necessary for this imaging study.

Fasting

We will ask you to fast for a minimum of 6 hours before the FDG study. We will check your blood sugar using blood from a finger stick before we inject the FDG. If your blood sugar is too low or if you feel like your blood sugar is too low at any time during the study, we can provide you with juice.

Radiation Risks

The ^{18}F-FDG is a radiotracer and therefore, you will be receiving a dose of radiation from your participation in this research. The maximum amount of radiation from the research-related radiation procedures in this study is equivalent to approximately 92% of the annual radiation limit for a medical worker. Long-term effects on your health, such as cancer, cannot be ruled out from this amount of radiation. This dose estimate takes into account only the exposure to research procedures in this project. If you have participated in other research studies involving radiation exposure, you should be aware that the risk of effects from radiation is believed to increase with each exposure you receive (including studies performed as part of your medical care).

Radiation Exposure in Women Capable of Becoming Pregnant

You may not participate in this study if you are pregnant. If you are capable of becoming pregnant, we will perform a pregnancy test before exposure to research-related radiation. You must tell us if you may have become pregnant within the previous 14 days because the pregnancy test is unreliable during that time.

Insertion of catheter (tube) into an arm vein (during PET scan)

A catheter (tube) will be inserted into a vein to allow us to give you the FDG. As in any procedure involving catheters placed in veins, there are several risks, including infection, clotting or continued bleeding. There may also be bruising, skin irritation and a dull ache at the site of insertion or a feeling of dizziness or fainting upon placement. These risks will be minimized by having only medical personnel trained in the placement of venous catheters inserting the line.

WHAT ARE THE BENEFITS OF THIS STUDY?

You will not benefit from being in this study. However, we hope that, in the future, other people might benefit from this study because this study is designed for the researchers to learn more about how tDCS may improve the treatment of the debilitating symptoms of a variety of diseases, including Multiple Sclerosis.

WILL IT COST ME ANYTHING TO BE IN THIS STUDY?

You will be responsible for any parking and/or transportation costs associated with your visits. You will not have any other costs for being in this research study. All procedures and tests will be provided at no cost to you. You and/or your medical/hospital insurance carrier will remain responsible for your regular medical care expenses.

WILL I BE PAID FOR PARTICIPATING?

You will be paid for being in this research study. You will receive $20 for the first visit, $40 for the second visit, and $40 for the third visit ($100 total).

WHO IS FUNDING THIS STUDY?

The University and the research team are receiving no payments from other agencies, organizations, or companies to conduct this research study.

WHAT IF I AM INJURED AS A RESULT OF THIS STUDY?

- If you are injured or become ill from taking part in this study, medical treatment is available at the University of Iowa Hospitals and Clinics.
- The University of Iowa does not plan to provide free medical care or payment for treatment of any illness or injury resulting from this study unless it is the direct result of proven negligence by a University employee.
- If you experience a research-related illness or injury, you and/or your medical or hospital insurance carrier will be responsible for the cost of treatment.

WILL YOU KEEP MY NAME ON FILE TO GIVE TO OTHERS?

We will keep information about you in a special kind of computer listing called a registry. A registry keeps information about you on file so that *other* researchers, not involved in this particular study, may contact you in the future about whether you are interested in being in *different* research studies. The registry will contain information such as your name, address, age, and selected other information such as gender, weight and height. We will keep the information in this registry secure by on a password-protected computer in a restricted-access office. You may request that your personal information be removed from this file at any time by contacting Thorsten Rudroff at (319) 467-0746 or by writing to him at E432 FH, University of Iowa, Iowa City Iowa 52242. If you choose to do this, your information will be permanently removed from our contact list.

WHAT ABOUT CONFIDENTIALITY?

We will keep your participation in this research study confidential to the extent permitted by law. However, it is possible that other people such as those indicated below may become aware of your participation in this study and may inspect and copy records pertaining to this research. Some of these records could contain information that personally identifies you.

- Federal government regulatory agencies,
- The U.S. Food and Drug Administration
- Auditing departments of the University of Iowa, and
- The University of Iowa Institutional Review Board (a committee that reviews and approves research studies)

To help protect your confidentiality, we will keep private all research records that identify you. Your data will be coded to remove any direct links to your identity, and any identifying material will be locked in a secure filing cabinet. If we write a report or article about this study or share the study data set with others, we will do so in such a way that you cannot be directly identified.

The University of Iowa Hospitals and Clinics generally requires that we document your participation in research occurring in a University of Iowa Health Care facility. This documentation will be in either your medical record or a database maintained on behalf of the institution reflecting that you are participating in this study. The information included will provide contact information for the research team as well as information about the risks associated with this study. We will keep this Informed Consent Document in our research files; it will not be placed in your medical record chart.

WILL MY HEALTH INFORMATION BE USED DURING THIS STUDY?

The Federal Health Insurance Portability and Accountability Act (HIPAA) requires University of Iowa Health Care to obtain your permission for the research team to access or create "protected health information" about you for purposes of this research study. Protected health information is information that personally identifies you and relates to your past, present, or future physical or mental health condition or care. We will access or create health information about you, as described in this document, for purposes of this research study. Once your health care provider has disclosed your protected health information to us, it may no longer be protected by the Federal HIPAA privacy regulations, but we will continue to protect your confidentiality as described under "Confidentiality."

We may share your health information related to this study with other parties including federal government regulatory agencies, the University of Iowa Institutional Review Boards and support staff.

You cannot participate in this study unless you permit us to use your protected health information. If you choose *not* to allow us to use your protected health information, we will discuss any non-research alternatives available to you. Your decision will not affect your right to medical care that is not research-related. Your signature on this Consent Document authorizes your health care provider to give us permission to use or create health information about you.

Although you may not be allowed to see study information until after this study is over, you may be given access to your health care records by contacting your health care provider. Your permission for us

to access or create protected health information about you for purposes of this study has no expiration date. You may withdraw your permission for us to use your health information for this research study by sending a written notice to Thorsten Rudroff, Department of Health & Human Physiology, University of Iowa, E432, Field House, Iowa City, Iowa 52242. However, we may still use your health information that was collected before withdrawing your permission. Also, if we have sent your health information to a third party, such as the study sponsor, or we have removed your identifying information, it may not be possible to prevent its future use. You will receive a copy of this signed document.

IS BEING IN THIS STUDY VOLUNTARY?

Taking part in this research study is completely voluntary. You may choose not to take part at all. If you decide to be in this study, you may stop participating at any time. If you decide not to be in this study, or if you stop participating at any time, you won't be penalized or lose any benefits for which you otherwise qualify.

What if I Decide to Drop Out of the Study?

Your participation in this research is voluntary. If you decide to participate, you may withdraw your consent and stop participating at any time.

Will I Receive New Information About the Study while Participating?

If we obtain any new information during this study that might affect your willingness to continue participating in the study, we'll promptly provide you with that information.

Can Someone Else End my Participation in this Study?

Under certain circumstances, the researchers might decide to end your participation in this research study earlier than planned. This might happen because in our judgment it would not be safe or comfortable for you to continue.

WHAT IF I HAVE QUESTIONS?

We encourage you to ask questions. If you have any questions about the research study itself, please contact: Thorsten Rudroff, (319) 467-0363. If you experience a research-related injury, please contact: Thorsten Rudroff, (319) 467-0363.

If you have questions, concerns, or complaints about your rights as a research subject or about research related injury, please contact the Human Subjects Office, 105 Hardin Library for the Health Sciences, 600 Newton Rd, The University of Iowa, Iowa City, IA 52242-1098, (319) 335-6564, or e-mail irb@uiowa.edu. General information about being a research subject can be found by clicking "Info for Public" on the Human Subjects Office web site, http://hso.research.uiowa.edu/. To offer input about your experiences as a research subject or to speak to someone other than the research staff, call the Human Subjects Office at the number above.

This Informed Consent Document is not a contract. It is a written explanation of what will happen during the study if you decide to participate. You are not waiving any legal rights by signing this Informed Consent Document. Your signature indicates that this research study has been explained to you, that your questions have been answered, and that you agree to take part in this study. You will receive a copy of this form.

FOR IRB USE ONLY
APPROVED BY: IRB-01
IRB ID #: 201905825
APPROVAL DATE: 11/18/19

University of Iowa
CONFIDENTIAL

Subject's Name (printed): [illegible]

University of Iowa
CONFIDENTIAL

[illegible] 7/13/2020

(Signature of Subject) (Date)

Statement of Person Who Obtained Consent

I have discussed the above points with the subject or, where appropriate, with the subject's legally authorized representative. It is my opinion that the subject understands the risks, benefits, and procedures involved with participation in this research study.

Alexandra [illegible] 7/13/2020

(Signature of Person who Obtained Consent) (Date)

FOR IRB USE ONLY
APPROVED BY: IRB-01
IRB ID #: 201905825
APPROVAL DATE: 11/18/19

INFORMED CONSENT DOCUMENT

Project Title: **Transcranial Direct Current Stimulation to reduce asymmetric glucose uptake in leg muscles of persons with Multiple Sclerosis**

Principal Investigator: Thorsten Rudroff, PhD

Research Team Contact:

Thorsten Rudroff, PhD	(319) 467-0363
Laura Ponto, PhD	(319) 356-1857
Craig D. Workman, PhD	(319) 467-0746

This consent form describes the research study to help you decide if you want to participate. This form provides important information about what you will be asked to do during the study, about the risks and benefits of the study, and about your rights as a research subject.

- If you have any questions about or do not understand something in this form, you should ask the research team for more information.
- You should discuss your participation with anyone you choose such as family or friends.
- Do not agree to participate in this study unless the research team has answered your questions and you decide that you want to be part of this study.

WHAT IS THE PURPOSE OF THIS STUDY?

This is a research study. We are inviting you to participate in this research study because you have been diagnosed with Multiple Sclerosis (MS).

The purpose of this research study is to investigate the effects of transcranial direct current stimulation (tDCS) on walking ability and the energy usage of the legs of people with MS. We will determine which of your legs is weaker with an isokinetic device. This specialized apparatus provides variable resistance to a movement, so that no matter how much effort you exert, the movement takes place at a constant speed. We will also administer either active tDCS (wearing the device with active stimulation) or sham tDCS (wearing the device without active stimulation) over the part of your brain that controls your weaker leg on two different days separated by at least 5 days.

tDCS is a non-invasive technique in which a very weak electrical current is applied to the head in order to stimulate the brain. To accomplish this, we will place electrodes soaked in salt water over your scalp and forehead. This means that one of the electrodes will be placed over your hair, but no additional substances will be put in your hair and no additional preparation of the hair is necessary. The electrodes will be held in place with a headband. A small current will be passed between the electrodes. You may feel some tingling under the electrodes when the current is first turned on, but no other adverse effects are expected. The stimulation will last for no longer than 10 min.

HOW MANY PEOPLE WILL PARTICIPATE?

Approximately 20 people will take part in this study conducted by investigators at the University of Iowa.

HOW LONG WILL I BE IN THIS STUDY?

If you agree to take part in this study, you will be asked to complete three total study sessions. Each session will be separated by at least 5 days. The first session, which will take place at the Integrative Neurophysiology Laboratory (INPL), will involve muscle strength testing and completing a few questionnaires. This visit is expected to take approximately 1 hour. Sessions 2 and 3, which will take place at the Positron Emission Tomography Imaging Center (PET Imaging Center) and the PET Imaging Lab at Pappajohn Biomedical Discovery Building, will involve walking on a treadmill for 20 minutes in conjunction with either active or sham tDCS. During this time, we will inject you with a radiotracer called [^{18}F]Fluorodeoxyglucose or FDG that will allow us to examine the energy use of your muscles during walking. After you walk on the treadmill, you will undergo PET imaging of your entire body. These sessions will take approximately 1.5 to 2 hours.

WHAT WILL HAPPEN DURING THIS STUDY?

Upon arrival to the INPL for your first session, the protocol procedures will be explained to you. If you agree to participate and sign this consent document, you will complete the Patient Determine Disease Steps and Fatigue Severity Scale questionnaires. You will then perform the leg strength assessment to determine your weaker leg. For Sessions 2 and 3, you will go to the PET Imaging Center and will be taken to the PET Imaging Lab at Pappajohn Biomedical Discovery Building. Next, we will place the two tDCS electrodes on your head; one over the part of your brain that controls your weaker leg and the other above your eyebrow on the other side of your head. Once these electrodes are secured in their place, we will randomly administer active (device on; 3 mA) or sham (device off; 0 mA) tDCS for 10 minutes. We will not tell you if the device is on or off. Once the stimulation is complete, you will remain seated for an additional 10 minutes before you walk on a treadmill for 20 minutes at a speed of your choice. You will walk at the same speed for both sessions and we will ask you to rate how much exertion (effort) you feel it took to complete the walk. Immediately after you have completed the walk, you will be positioned in the PET scanner and a full-body scan will be performed to determine how the muscles of your body have used energy (glucose) during the walk.

PET Scans

This study involves an imaging technique called positron emission tomography (PET). In this scan, the PET scanner takes pictures of the function of your brain and muscles using a radioactive form of sugar called [^{18}F]fluorodeoxyglucose or FDG to determine which parts of the body are most active.

For sessions 2 and 3, the same procedures will be done. You will need to fast for a minimum of 6 hours before the FDG scan.

When you first arrive at the PET Imaging Center, we will measure your height and weight while clothed. On the day that you are going to have the FDG imaging, we will check your blood sugar to make sure that it is at a level of 200 mg/dL or lower. If your blood sugar is higher than 200 mg/dL, we will not be able to do the FDG scan on that day. If you are able to proceed with the scan a small thin tube (catheter) will be placed in a vein that will be used for injecting the PET radiotracer, FDG. We will take you over to the PET lab in the Pappajohn Biomedical Discovery Building where you will be asked to use the restroom and empty your bladder before you will walk on the treadmill and be imaged in the PET scanner.

The PET scanner is a large circular ring of detectors that forms a donut shape. You will be lying on a

FOR IRB USE ONLY
APPROVED BY: IRB-01
IRB ID #: 201905825
APPROVAL DATE: 11/18/19

bed that can be moved into the middle of this donut during imaging (i.e., taking the pictures). Initially, we will take a CT image to use to correct the subsequent PET images and then we will take the PET images of your entire body (top of your head to your toes). It is important that you lie quietly and not move during imaging.

When the imaging is complete, we will remove the IV catheter and ask you to go to the restroom again before you leave the PET Imaging Center. Each PET scan will last approximately 2 hours.

Tissue/Blood/Data Storage for Future Use

As part of this study, we are obtaining PET images from you. We will study your PET images in the future, after this study is over. Your sample, information, and/or data may be placed in a central repository or other national repositories sponsored by the National Institutes of Health or other Federal agencies. If this happens, it may be stripped of identifiers (such as name, date of birth, address, etc). Other qualified researchers who obtain proper permission may gain access to your sample and/or data for use in approved research studies that may or may not be related to in the purpose of this study.

The tests we might want to use to study your PET images may not even exist at this time. Therefore, we are asking for your permission to store your PET images so that we can study them in the future. These future studies may provide additional information that will be helpful in understanding Multiple Sclerosis, but it is unlikely that what we learn from these studies will have a direct benefit to you. It is possible that your PET images might be used to develop products tests, or discoveries that could be patented and licensed. In some instances, these may have potential commercial value and may be developed by the Investigators, University of Iowa, commercial companies, organizations funding this research, or others that may not be working directly with this research team. However, donors of PET images do not retain any property rights to the materials. Therefore, there are no plans to provide financial compensation to you should this occur.

Your PET images will be stored *with a code which may be linked to* your age (date of birth), height, weight, Multiple Sclerosis diagnosis, and the date the images were obtained. If you agree now to future use of your PET images but decide in the future that you would like to have it removed from future research, you should contact Dr. Thorsten Rudroff by phone at 319-467-0363. However, if some research with your PET images has already been completed, the information from that research may still be used.

WILL I BE NOTIFIED IF MY IMAGES RESULT IN AN UNEXPECTED FINDING?

The results from the images we collect in this research study are not the same quality as what you would receive as part of your routine health care. The image results will not be reviewed by a physician who normally reads such results. Due to this, you will not be informed of any unexpected findings. The results of your images will not be placed in your medical record with your primary care physician or otherwise. If you believe you are having symptoms that may require care, you should contact your primary care physician.

WHAT ARE THE RISKS OF THIS STUDY?

You may experience one or more of the risks indicated below from being in this study. In addition to

these, there may be other unknown risks, or risks that we did not anticipate, associated with being in this study.

Risks of tDCS

tDCS has been conducted on humans and animals for many years and no evidence has emerged to suggest that it is harmful or has ever induced a serious side effect. However, the safety of tDCS is dependent upon current strength, electrode size, and stimulation duration. We will follow the existing guidelines for safe and effective stimulation that have been identified for research studies and in clinical practice for tDCS applications. In the several hundred studies conducted on humans using tDCS, the only side effects that have been reported when proper guidelines are followed are temporary tingling, itching, mild headache, or skin redness under the electrodes in some subjects.

Risks of PET Imaging

This study involves the administration of a PET radiopharmaceutical, [^{18}F]fluorodeoxyglucose or FDG. FDG has been used at the University of Iowa and throughout the world with no known adverse reactions reported in the medical literature. The only discomfort during the PET scans will be from lying still in the confined space of the scanner for the required length of time, fasting for 6 hours, and that associated with the venous catheter necessary for this imaging study.

Fasting

We will ask you to fast for a minimum of 6 hours before the FDG study. We will check your blood sugar using blood from a finger stick before we inject the FDG. If your blood sugar is too low or if you feel like your blood sugar is too low at any time during the study, we can provide you with juice.

Radiation Risks

The ^{18}F-FDG is a radiotracer and therefore, you will be receiving a dose of radiation from your participation in this research. The maximum amount of radiation from the research-related radiation procedures in this study is equivalent to approximately 92% of the annual radiation limit for a medical worker. Long-term effects on your health, such as cancer, cannot be ruled out from this amount of radiation. This dose estimate takes into account only the exposure to research procedures in this project. If you have participated in other research studies involving radiation exposure, you should be aware that the risk of effects from radiation is believed to increase with each exposure you receive (including studies performed as part of your medical care).

Radiation Exposure in Women Capable of Becoming Pregnant

You may not participate in this study if you are pregnant. If you are capable of becoming pregnant, we will perform a pregnancy test before exposure to research-related radiation. You must tell us if you may have become pregnant within the previous 14 days because the pregnancy test is unreliable during that time.

Insertion of catheter (tube) into an arm vein (during PET scan)

A catheter (tube) will be inserted into a vein to allow us to give you the FDG. As in any procedure involving catheters placed in veins, there are several risks, including infection, clotting or continued bleeding. There may also be bruising, skin irritation and a dull ache at the site of insertion or a feeling of dizziness or fainting upon placement. These risks will be minimized by having only medical personnel trained in the placement of venous catheters inserting the line.

WHAT ARE THE BENEFITS OF THIS STUDY?

You will not benefit from being in this study. However, we hope that, in the future, other people might benefit from this study because this study is designed for the researchers to learn more about how tDCS may improve the treatment of the debilitating symptoms of a variety of diseases, including Multiple Sclerosis.

WILL IT COST ME ANYTHING TO BE IN THIS STUDY?

You will be responsible for any parking and/or transportation costs associated with your visits. You will not have any other costs for being in this research study. All procedures and tests will be provided at no cost to you. You and/or your medical/hospital insurance carrier will remain responsible for your regular medical care expenses.

WILL I BE PAID FOR PARTICIPATING?

You will be paid for being in this research study. You will receive $20 for the first visit, $40 for the second visit, and $40 for the third visit ($100 total).

WHO IS FUNDING THIS STUDY?

The University and the research team are receiving no payments from other agencies, organizations, or companies to conduct this research study.

WHAT IF I AM INJURED AS A RESULT OF THIS STUDY?

- If you are injured or become ill from taking part in this study, medical treatment is available at the University of Iowa Hospitals and Clinics.
- The University of Iowa does not plan to provide free medical care or payment for treatment of any illness or injury resulting from this study unless it is the direct result of proven negligence by a University employee.
- If you experience a research-related illness or injury, you and/or your medical or hospital insurance carrier will be responsible for the cost of treatment.

WILL YOU KEEP MY NAME ON FILE TO GIVE TO OTHERS?

We will keep information about you in a special kind of computer listing called a registry. A registry keeps information about you on file so that *other* researchers, not involved in this particular study, may contact you in the future about whether you are interested in being in *different* research studies. The registry will contain information such as your name, address, age, and selected other information such as gender, weight and height. We will keep the information in this registry secure by on a password-protected computer in a restricted-access office. You may request that your personal information be removed from this file at any time by contacting Thorsten Rudroff at (319) 467-0746 or by writing to him at E432 FH, University of Iowa, Iowa City Iowa 52242. If you choose to do this, your information will be permanently removed from our contact list.

FOR IRB USE ONLY
APPROVED BY: IRB-01
IRB ID #: 201905825
APPROVAL DATE: 11/18/19

WHAT ABOUT CONFIDENTIALITY?

We will keep your participation in this research study confidential to the extent permitted by law. However, it is possible that other people such as those indicated below may become aware of your participation in this study and may inspect and copy records pertaining to this research. Some of these records could contain information that personally identifies you.

- Federal government regulatory agencies,
- The U.S. Food and Drug Administration
- Auditing departments of the University of Iowa, and
- The University of Iowa Institutional Review Board (a committee that reviews and approves research studies)

To help protect your confidentiality, we will keep private all research records that identify you. Your data will be coded to remove any direct links to your identity, and any identifying material will be locked in a secure filing cabinet. If we write a report or article about this study or share the study data set with others, we will do so in such a way that you cannot be directly identified.

The University of Iowa Hospitals and Clinics generally requires that we document your participation in research occurring in a University of Iowa Health Care facility. This documentation will be in either your medical record or a database maintained on behalf of the institution reflecting that you are participating in this study. The information included will provide contact information for the research team as well as information about the risks associated with this study. We will keep this Informed Consent Document in our research files; it will not be placed in your medical record chart.

WILL MY HEALTH INFORMATION BE USED DURING THIS STUDY?

The Federal Health Insurance Portability and Accountability Act (HIPAA) requires University of Iowa Health Care to obtain your permission for the research team to access or create "protected health information" about you for purposes of this research study. Protected health information is information that personally identifies you and relates to your past, present, or future physical or mental health condition or care. We will access or create health information about you, as described in this document, for purposes of this research study. Once your health care provider has disclosed your protected health information to us, it may no longer be protected by the Federal HIPAA privacy regulations, but we will continue to protect your confidentiality as described under "Confidentiality."

We may share your health information related to this study with other parties including federal government regulatory agencies, the University of Iowa Institutional Review Boards and support staff.

You cannot participate in this study unless you permit us to use your protected health information. If you choose *not* to allow us to use your protected health information, we will discuss any non-research alternatives available to you. Your decision will not affect your right to medical care that is not research-related. Your signature on this Consent Document authorizes your health care provider to give us permission to use or create health information about you.

Although you may not be allowed to see study information until after this study is over, you may be given access to your health care records by contacting your health care provider. Your permission for us

to access or create protected health information about you for purposes of this study has no expiration date. You may withdraw your permission for us to use your health information for this research study by sending a written notice to Thorsten Rudroff, Department of Health & Human Physiology, University of Iowa, E432, Field House, Iowa City, Iowa 52242. However, we may still use your health information that was collected before withdrawing your permission. Also, if we have sent your health information to a third party, such as the study sponsor, or we have removed your identifying information, it may not be possible to prevent its future use. You will receive a copy of this signed document.

IS BEING IN THIS STUDY VOLUNTARY?

Taking part in this research study is completely voluntary. You may choose not to take part at all. If you decide to be in this study, you may stop participating at any time. If you decide not to be in this study, or if you stop participating at any time, you won't be penalized or lose any benefits for which you otherwise qualify.

What if I Decide to Drop Out of the Study?
Your participation in this research is voluntary. If you decide to participate, you may withdraw your consent and stop participating at any time.

Will I Receive New Information About the Study while Participating?
If we obtain any new information during this study that might affect your willingness to continue participating in the study, we'll promptly provide you with that information.

Can Someone Else End my Participation in this Study?
Under certain circumstances, the researchers might decide to end your participation in this research study earlier than planned. This might happen because in our judgment it would not be safe or comfortable for you to continue.

WHAT IF I HAVE QUESTIONS?

We encourage you to ask questions. If you have any questions about the research study itself, please contact: Thorsten Rudroff, (319) 467-0363. If you experience a research-related injury, please contact: Thorsten Rudroff, (319) 467-0363.

If you have questions, concerns, or complaints about your rights as a research subject or about research related injury, please contact the Human Subjects Office, 105 Hardin Library for the Health Sciences, 600 Newton Rd, The University of Iowa, Iowa City, IA 52242-1098, (319) 335-6564, or e-mail irb@uiowa.edu. General information about being a research subject can be found by clicking "Info for Public" on the Human Subjects Office web site, http://hso.research.uiowa.edu/. To offer input about your experiences as a research subject or to speak to someone other than the research staff, call the Human Subjects Office at the number above.

This Informed Consent Document is not a contract. It is a written explanation of what will happen during the study if you decide to participate. You are not waiving any legal rights by signing this Informed Consent Document. Your signature indicates that this research study has been explained to you, that your questions have been answered, and that you agree to take part in this study. You will receive a copy of this form.

FOR IRB USE ONLY
APPROVED BY: IRB-01
IRB ID #: 201905825
APPROVAL DATE: 11/18/19

University of Iowa
CONFIDENTIAL

Subject's Name (printed): ____________________

University of Iowa
CONFIDENTIAL

____________________ 7/9/2020
(Signature of Subject) (Date)

Statement of Person Who Obtained Consent

I have discussed the above points with the subject or, where appropriate, with the subject's legally authorized representative. It is my opinion that the subject understands the risks, benefits, and procedures involved with participation in this research study.

Alexandra [illegible] 7/13/2020
(Signature of Person who Obtained Consent) (Date)
